# Bidirectionally Thermochromic Nanocolloid System for on‐Demand Optical Switching and Agricultural Energy Management

**DOI:** 10.1002/advs.202519759

**Published:** 2025-11-12

**Authors:** Qinbo Jiang, Jiayi Li, Hui Zhang

**Affiliations:** ^1^ College of Biosystems Engineering and Food Science Zhejiang University Hangzhou 310058 China

**Keywords:** bidirectional thermochromism, cononsolvency, nanocolloid, sustainable agriculture, thermochromic smart window

## Abstract

Extreme weather and massive energy demands in facility agriculture threaten food security. However, the current optical switching strategy fails to provide adequate crop climate management, since creating thermochromic materials capable of reversible and temperature‐bidirectional optical modulation across a wide temperature range remains a formidable challenge. Here, a nanocolloid system comprising two tailored thermoresponsive copolymers that achieve optical‐thermal regulation by a temperature‐bidirectional phase transition is reported. Adopting the cononsolvency in a binary solvent, the nanocolloid exhibits a widely tunable transition from 27–85 °C (heat‐induced) and −9–36 °C (cold‐induced). In the transparent state, the nanocolloid‐based smart window achieves a high photosynthetically active radiation (PAR) transmittance (>91%). Upon heating, it shows remarkable solar modulation ability (*ΔT_sol_* up to 65.43%), while upon cooling, it provides a high PAR diffuse reflectance of 27.92% and enhances supplemental lighting efficiency by 33.91%. As a proof of concept in climate‐resilient agriculture, nanocolloid‐based smart windows reduce energy consumption by 11.61 kJ·m^−3^ (cooling) and 2.96 kJ·m^−3^ (heating), while boosting photosynthetic rates of specific crops by 222.19% and 126.07% under heat and cold stress, respectively. The bidirectional optical‐thermal regulation by nanocolloid‐based smart windows enables low‐energy agriculture through higher light use efficiency, thereby reducing the carbon footprint in sustainable agriculture.

## Graphical abstract

A bidirectionally thermochromic nanocolloid system is developed for facility agriculture. The bidirectional and wide‐temperature response dynamically modulates light radiation, stabilizing the indoor climate, improving supplemental lighting efficiency, and accelerating plant photosynthesis. By passively adapting to environmental changes, the smart window is expected to markedly reduce the electricity demand of agricultural facilities and support low‐carbon farming.

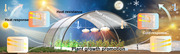

## 1 Introduction

**Figure 1:**
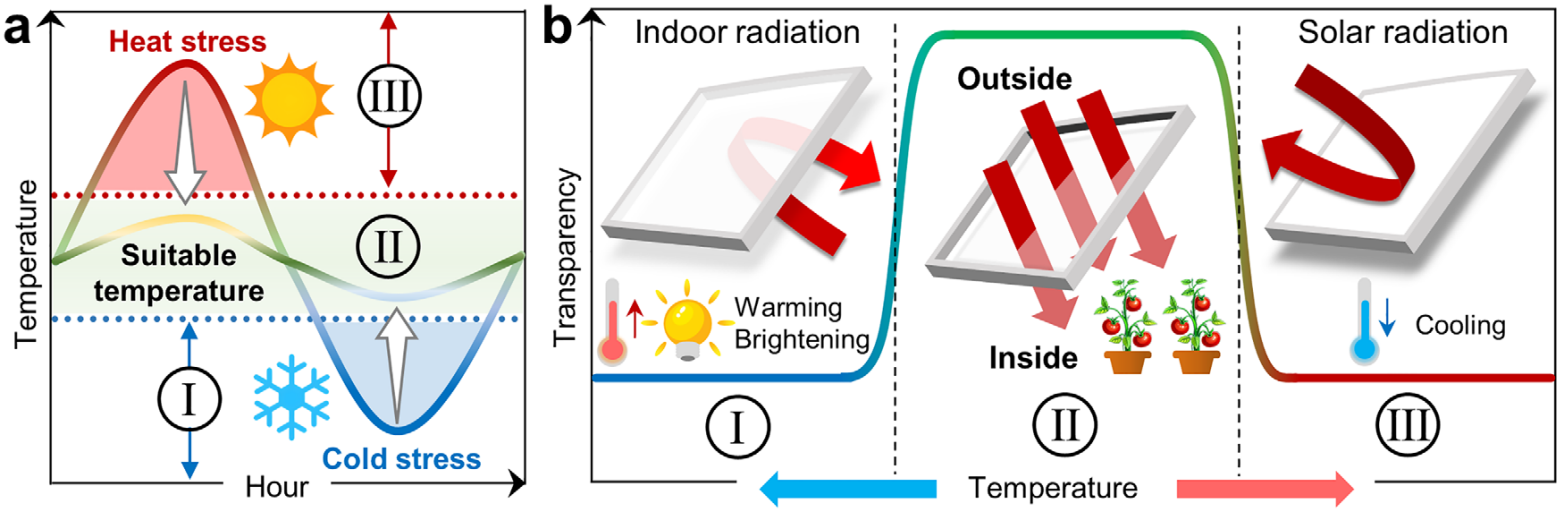
Proposed regulation of temperature for indoor agriculture. a) Reducing the diurnal temperature range is the key to maintaining the stability of environments, that is, to increase the daily low temperature (I) and decrease the daily high temperature (III), ultimately making the temperature fluctuate within the appropriate range (II). b) Proposed management of temperature and light by smart windows includes cold‐induced thermochromism for warming and brightening in a cold environment (I) and heat‐induced thermochromism for cooling in a hot environment (III), while sunlight can enter through the window normally when the temperature is suitable (II).

In electro‐agriculture, including greenhouse and vertical farming systems,^[^ ^1^ ^]^ energy consumption for stabilizing the environment of crops accounts for 78% of total energy expenditure.^[^ ^2^ ^]^ For decarbonization of the agricultural sector, which is responsible for ≈15% of global CO_2_ emissions ^[^ ^3^, ^4^, ^5^ ^],^ the smart optical switching strategy has great potential in effectively regulating thermal radiation.^[^ ^6^, ^7^, ^8^ ^]^ Compared with mechanical force, electricity, and light‐induced optical switching strategies, thermally responsive switching materials stand out for requiring no additional energy consumption.^[^ ^9^, ^10^ ^]^ Thermally responsive materials capable of dynamically and reversibly modulating the optical properties in response to external stimuli are a central pursuit in the optical switching strategy of smart windows.^[^ ^11^ ^]^ Particularly formidable is the challenge of achieving bidirectional response to both heating and cooling within a single system across a broad temperature range. Such a capability can enable precise, autonomous control over solar radiation and thermal energy, which is crucial for energy saving. Especially, growth conditions (sunlight and temperature) of various crops are quite different, requiring smart windows with extensive responses to assist crops in growing in hot or cold environments by regulating photosynthetic radiation and temperature (**Figure** **1**). However, simultaneously integrating a wide response range, high optical regulation, and bidirectional response remains a difficult goal.

In thermochromic optical switching materials for smart windows, the typical VO_2_ has a comparatively high transition temperature (*T_c_*) of ≈68 °C.^[^ ^12^ ^]^ Although doping with the W element can significantly reduce the *T_c_* of VO_2_ nanoparticles to below 40 °C, the optical performance will be sacrificed as the W element increases.^[^ ^13^, ^14^, ^15^ ^]^ The optical performance of metal oxide‐based thermochromic materials is weak, with the luminous transmittance (*T_lum_*) and sunlight regulation (*ΔT_sol_*), respectively, less than 70% and 50% even after being improved by the design of nanoarrays and doping of inorganic nanoparticles.^[^ ^16^, ^17^ ^]^ The perovskite is also a widely studied thermochromic material,^[^ ^18^ ^]^ depending on the thermal energy‐driven reaction with gases (such as H_2_O and NH_3_).^[^ ^19^, ^20^ ^]^ However, the thermally driven structural switching of perovskites always leads to conspicuous colors with comparatively weak *ΔT_sol_* (<40%), potentially limiting the application in smart windows.^[^ ^21^, ^22^ ^]^ Thermochromic gels (hydrogels, organohydrogels, and ionogels) show outstanding performances of sunlight regulation, the suitable thermochromic temperature for the human body, and the constant white after thermochromism.^[^ ^23^, ^24^, ^25^ ^]^ The thermochromism of gels relies on the critical solution of molecules constituting the network.^[^ ^26^ ^]^ When the temperature exceeds the critical solution temperature, the interaction between the network and mediums will switch to intramolecular interaction (hydrogen bond, hydrophobic interaction, and beyond) to change transparency.^[^ ^27^ ^]^ Typical hydrogels with heat‐induced chromism are based on copolymers of *N*‐isopropylacrylamide (NIPAM), with ≈30 °C of a lower critical solution temperature (LCST) in water.^[^ ^28^ ^]^ P(NIPAM) hydrogels have remarkable optical performances, with *T_lum_* and *ΔT_sol_* exceeding 90% and 88%, respectively.^[^ ^29^ ^]^ Similarly, the upper critical solution temperature (UCST, >20 °C) of zwitterionic hydrogels can make smart windows with cold‐induced chromism, with a moderate *T_lum_* of 37.1% and a high annual energy‐saving of 30.7%.^[^ ^30^ ^]^ Sodium dodecyl sulfate/potassium tartrate micelles have been reported to act as cold‐induced chromic materials and penetrate into p(NIPAM) hydrogels to prepare bidirectional‐thermochromism smart windows, with the UCST of 9.5–25.5 °C and LCST of 25.5–50 °C.^[^ ^31^ ^]^ However, the low efficiency of the penetration strategy, the narrow range of the thermochromic temperature, and the poor stability of the aqueous medium limit its application. Hence, an all‐in‐one optical switching strategy to realize extensive and bidirectional thermochromism is critical for developing smart windows with expanded applications.

Here, we report a bidirectionally and extensively thermochromic nanocolloid system based on organo‐hydrocolloids, aiming to develop an on‐demand optical switching strategy for smart windows with outstanding optical performances and strong environmental stability. The medium of nanocolloids is an organic solution composed of highly transparent PEG400 and water. Hydrogen bonds between PEG and water result in the cononsolvency for widely regulating the thermochromic temperature of smart windows and the freezing resistance for improving environmental stability. The dispersed phase consists of two copolymers with independent LCST and UCST: The NIPAM and *N*,*N*‐dimethylacrylamide (DMAA) polymerized p(NIPAM‐co‐DMAA); acrylic acid (AC) and acrylamide (AM) polymerized p(AC‐co‐AM). In nanocolloids, p(NIPAM‐co‐DMAA) and p(AC‐co‐AM) have a wide thermochromic range (LCST: 27–85 °C, UCST: −9–36 °C). In the transparent state, the *T_lum_*, transmittance of the sunlight (*T_sol_*), and transmittance of the photosynthetically active radiation (*T_PAR_*, 350–750 nm) of smart windows reach 92.31%, 75.51%, and 91.18%, respectively. On a summer day with a maximum air temperature of 39.2 °C, the *ΔT_sol_* of the smart window can reach 65.43% with 11.61 kJ·m^−3^ of the energy saving; a decrease of 4.4 °C in the highest leaf temperature of tomato seedlings under full sunlight can ultimately increase the CO_2_ assimilation by 222.19%. At an ambient temperature of −20 °C, the diffuse reflectance of the photosynthetically active radiation (*D_PAR_*) by smart windows can reach 27.92%. This results in 2.96 kJ·m^−3^ of energy savings for temperature regulation and a 35.73% increase in the PAR intensity of the supplementary light for indoor farming, thereby comprehensively increasing the photosynthetic rate by 126.07% in *Epipremnum aureum*. After 100 cycles (100 days) between −20 and 80 °C, the optical performances of smart windows remained stable with a fluctuation of less than 2%, demonstrating outstanding cyclic usability. The freezing point below −20 °C enables the smart window to maintain stability in extremely cold regions. Furthermore, we elucidate the underlying interactions, including the role of the Hofmeister series of ions in tuning the transition temperatures. This study systematically details the material design, mechanistic understanding, optical performance, energy‐saving evaluation, and agricultural efficacy of the proposed colloid‐based smart windows.

## 2 Results and Discussion

### 2.1 Bidirectional Thermochromism

**Figure 2:**
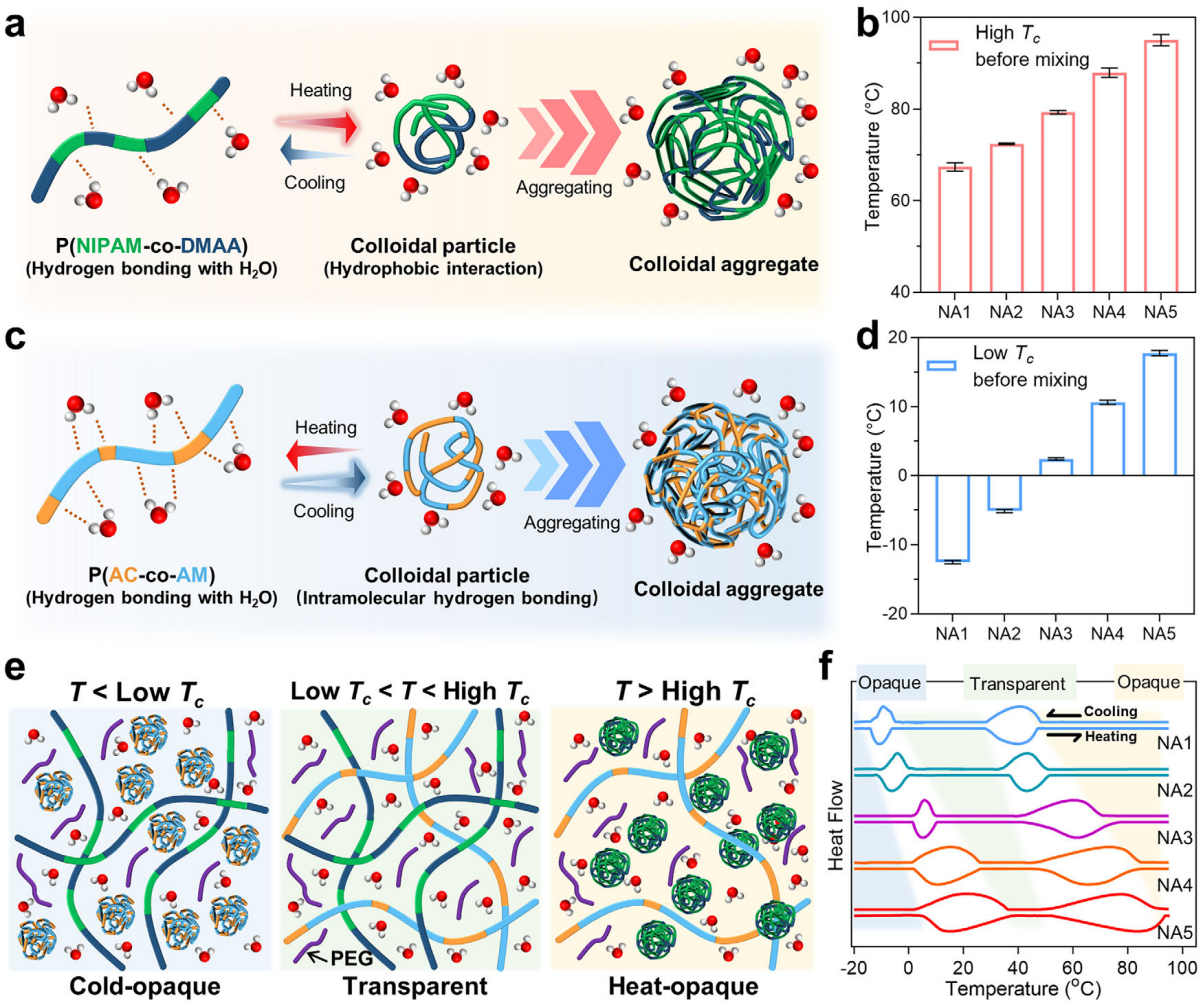
Composition and bidirectional thermochromism of nanocolloids. a) In heat‐induced thermochromism, hydrophobic interaction within p(NIPAM‐co‐DMAA) chains leads to the aggregation of copolymers, which form colloidal aggregates. b) The high *T_c_* of p(NIPAM‐co‐DMAA) from NA1 to NA5 before mixing with p(AC‐co‐AM). c) In the cold‐induced thermochromism, hydrogen bonding within p(AC‐co‐AM) chains replaces that between the copolymer and water during cooling, forming colloidal particles that gradually aggregate to form colloidal aggregates. d) The low *T_c_* of p(AC‐co‐AM) from NA1 to NA5 before mixing with p(NIPAM‐co‐DMAA). e) Schematic representation of mixed organo‐hydrocolloids with p(AC‐co‐AM) and p(NIPAM‐co‐DMAA) at *T*<low *T_c_*, low *T_c_* <*T*<high *T_c_*, and *T*>high *T_c_*, respectively. f) The heat flow of organo‐hydrocolloids in the heating‐cooling cycle of differential scanning calorimetry.

The independent syntheses of p(NIPAM‐co‐DMAA) and p(AC‐co‐AM) in an aqueous solution of PEG are described in Figure S1 and Table S1 (Supporting Information). The selective mixing of p(NIPAM‐co‐DMAA) and p(AC‐co‐AM) produces the nanocolloids for smart windows (NA1–NA5), with the inclusion of salts being optional. Polyethylene glycol (PEG400) is a highly transparent and widely used raw material in the chemical industry.^[^ ^32^ ^]^ The abundant PEG ethers (C–O–C) are acceptors for the hydrogen bonding of water molecules.^[^ ^33^ ^]^ These hydrogen bonds can increase the freeze resistance of PEG aqueous solutions and result in cononsolvency, greatly changing the critical solution temperature of copolymers.^[^ ^34^, ^35^ ^]^ We have reported the effectively reduced LCST of p(NIPAM) from 33 °C to <−25 °C by PEG.^[^ ^25^ ^]^ In nanocolloids, p(NIPAM‐co‐DMAA) and p(AC‐co‐AM) show bidirectional thermochromism by LCST and UCST, respectively. For the heat‐induced thermochromism, the stability of hydrogen bonds between p(NIPAM‐co‐DMAA) and water will decline, while the hydrophobic groups (isopropyl and dimethyl) will undergo hydrophobic interactions to separate out copolymers and generate colloidal particles (**Figure** **2**a; Figure S2a, Supporting Information).^[^ ^28^ ^]^ When the temperature drops below UCST, the hydrogen bonds between p(AC‐co‐AM) and water will be replaced by intramolecular hydrogen bonds among amide and carboxyl groups, causing aggregation of polymer chains for cold‐induced thermochromism (Figure 2c; Figure S2b, Supporting Information).^[^ ^30^ ^]^ Eventually, thermo‐induced colloidal particles will aggregate into micro/nano‐sized aggregates that are the basic unit for reflecting radiation, distributed in 100–4000 nm for p(NIPAM‐co‐DMAA) and 100–2000 nm for p(AC‐co‐AM), respectively (Figure S3, Supporting Information). Compared to the mold‐casting method (such as hydrogel‐based smart windows) ^[^ ^36^ ^],^ liquid nanocolloids can be easily filled into the interlayer of smart windows (Figure S4, Supporting Information), which makes the synthesis of thermochromic materials independent of the whole smart windows.

The temperature range of transparent states can be adjusted by a customized selection of p(NIPAM‐co‐DMAA) and p(AC‐co‐AM), thus achieving widely thermochromic performances for various growth environments of crops. For systematically investigating the regulation of thermochromic temperatures, we have studied the effects of ratios of PEG, proportions of monomers, additions of salts, and the mutual interference between p(NIPAM‐co‐DMAA) and p(AC‐co‐AM) on smart windows based on nanocolloids. Figures S5a and S6 (Supporting Information) detail the preponderant factor of PEG in widely regulating the *T_c_* of copolymers. The *T_c_* of the p(NIPAM‐co‐DMAA) in the aqueous solution will rapidly decrease from 39.1 to 1.6 °C as PEG increases to 50%, while the *T_c_* of the p(AC‐co‐AM) will increase from −16.4 to 5.4 °C. The cononsolvency of PEG aqueous solutions enables the instability of hydrogen bonding between copolymers and water molecules ^[^ ^25^ ^],^ resulting in the premature breakage of the hydrogen bonds during heating or cooling. The copolymerization of monomers with more hydrophilic features can enhance the interaction between copolymers and water (Figure S5b, Supporting Information).^[^ ^37^ ^]^ An equal mass of DMAA with NIPAM can increase the *T_c_* of p(NIPAM‐co‐DMAA) by 26 °C.

Figure 2b,d, and f and Table S2 (Supporting Information) illustrate the *T_c_* of two copolymers before and after mixing. The high *T_c_* of the p(NIPAM‐co‐DMAA) significantly decreases after being mixed with the p(AC‐co‐AM), with a maximum decrease of 28.8 °C (NA1). However, the low *T_c_* of the p(AC‐co‐AM) only changes slightly after mixing, confirming that the p(AC‐co‐AM) can affect the interaction of the p(NIPAM‐co‐DMAA) and water. Figure S7 (Supporting Information) shows the solvation free energy of the dimer fragment of NIPAM‐DMAA and AC‐AM by DFT. At −20, 20, and 95 °C, the solvation free energy of AC‐AM consistently exceeds that of NIPAM‐DMAA by more than 100%, which indicates that the p(AC‐co‐AM) should have the comparatively stronger hydrophilicity than that of the p(NIPAM‐co‐DMAA) due to carboxyl and amide groups. Therefore, the stability of the hydrogen bonding between p(NIPAM‐co‐DMAA) and water can be weakened by the p(AC‐co‐AM), further lowering the high *T_c_* of p(NIPAM‐co‐DMAA).

The effect of salts on thermochromic temperatures is displayed in Figure S8 (Supporting Information). Here, we report the 8 salts, including 2 cations (K^+^ and Na^+^) and 4 anions (SO_4_^2−^, Cl^−^, Br^−^, and I^−^). As described in the Hofmeister series, the chaotropic ability, which can promote the interaction between water and solutes, should be in the order of K^+^<Na^+^ and SO_4_^2−^<Cl^−^<Br^−^<I^−^.^[^ ^38^ ^]^ In the heat‐induced thermochromism, the *T_c_* is most sensitive to chaotropic NaI and kosmotropic K_2_SO_4_ (0.2 mol·L^−1^), showing the most significant increase (2.4 °C) and decrease (47.5 °C) that can individually be explained by the salt‐in and salt‐out effects.^[^ ^39^ ^]^ Negative hydration of chaotropic salts (I^−^) can enhance the stability of hydrogen bonds between p(NIPAM‐co‐DMAA) and water,^[^ ^40^ ^]^ thus increasing the *T_c_*. However, the decreased *T_c_* of the p(NIPAM‐co‐DMAA) caused by the other 6 salts (Br^−^, Cl^−^, SO_4_^2−^) enables the kosmotropic ability to be dominant. In the cold‐induced thermochromism, the effects of salts on the *T_c_* of the p(AC‐co‐AM) dispersed in the aqueous solvent of PEG show a sequence completely opposite to that in the p(AC‐co‐AM) dispersed in pure water.^[^ ^41^ ^]^ The inverse Hofmeister series possibly results from the synergy of the cononsolvency by PEG. The metastable hydration shell generated by the hydrogen bonding between PEG and water will be preferentially consolidated by chaotropic NaI,^[^ ^42^ ^]^ thus enhancing the effect of cononsolvency that will increase the *T_c_* of the p(AC‐co‐AM). The key evidence is the peak of salts with Cl^−^ at 0.2 mol·L^−1^, which may be caused by the switching of dominant roles between the salt‐induced indirect effect on the cononsolvency of PEG and the salt‐induced direct chaotropic ability on the p(AC‐co‐AM). In short, the salt effect is also an optional solution to regulate the high and low *T_c_* of nanocolloids, but the contribution of the salt type and concentration in the complex system needs to be considered.

In addition to the bidirectional thermochromism, the nanocolloids have an extensive thermochromic range, including the low and high *T_c_* of −9 to 36 °C and 27 to 85 °C, respectively, with the adjustable transparent range of −5 to 50 °C. The temperature range of the heat‐induced thermochromism in nanocolloids covers widely reported thermochromic materials (Figure S9, Supporting Information), such as metal oxides (≈ 50–80 °C),^[^ ^17^, ^43^, ^44^, ^45^ ^]^ perovskites (≈ 45–80 °C),^[^ ^18^, ^22^, ^46^, ^47^ ^]^ ionogels (≈ 30–50 °C),^[^ ^48^, ^49^, ^50^ ^]^ and hydrogels (≈ 10–50 °C).^[^ ^51^, ^52^, ^53^, ^54^ ^]^

### 2.2 Optical Performances

**Figure 3:**
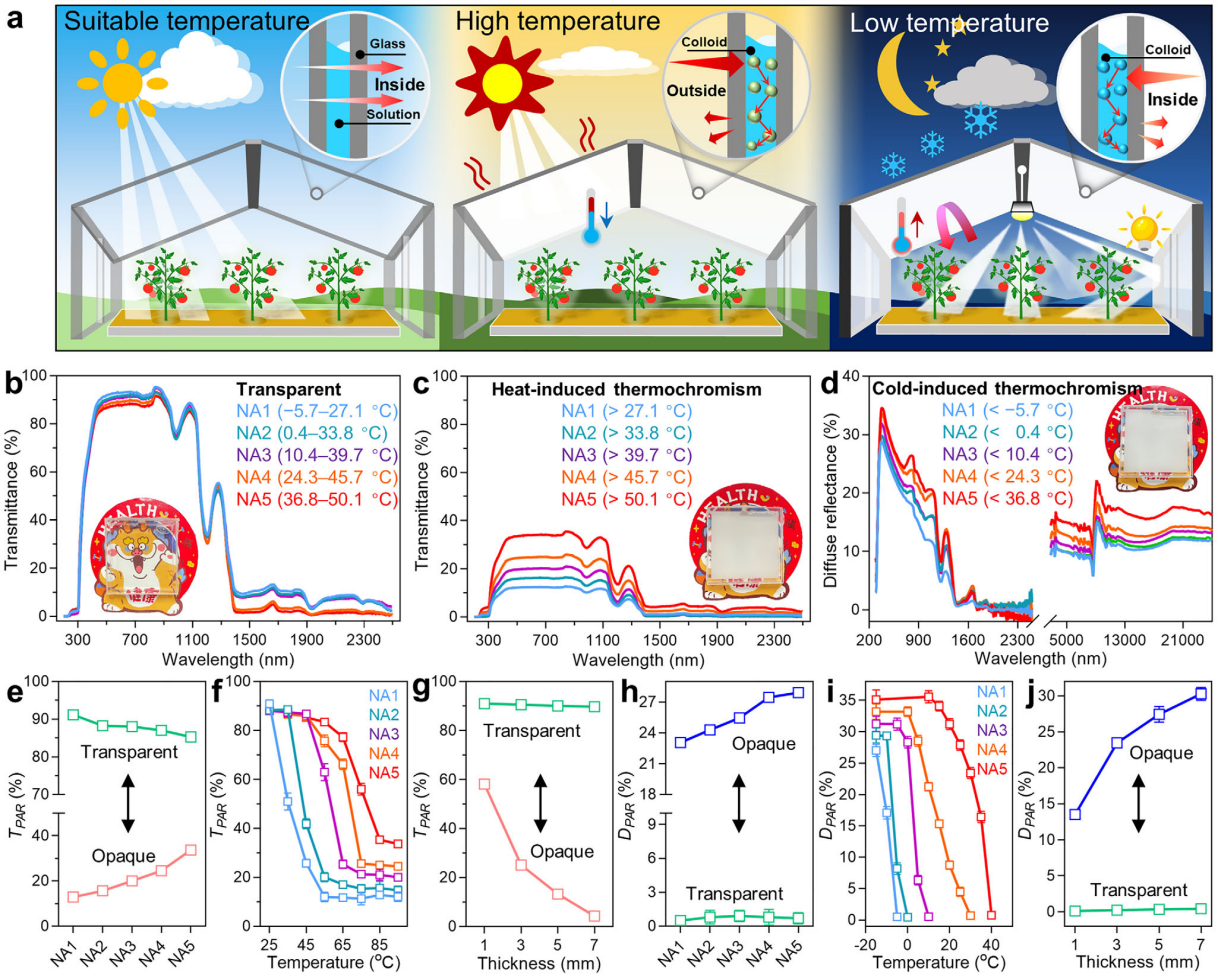
All‐climate management of light and temperature based on the optical performances of smart windows. a) Diagram of all‐climate management of radiation and temperature by smart windows: Sunlight can pass through the transparent smart window when the temperature is suitable for crop growth; sunlight can be diffused by the heat‐opaque smart window when the temperature is excessively high, cooling the indoor environment; the thermal radiation and the light emitted by the plant supplementary light can be locked indoors by the cold‐opaque smart window when the temperature is excessively low, thereby warming and brightening the environment. b,c) The transmittance of 3‐mm‐thickness smart windows during 250–2500 nm at the transparent state and heat‐opaque state. d) Diffuse reflectance of smart windows at cold‐opaque for visible light, near‐infrared, and mid‐infrared. e–g) *T_PAR_* of smart windows at transparent and opaque states, at different temperatures, and at different thicknesses. h–j) *D_PAR_* of smart windows at transparent and opaque states, at different temperatures, and at different thicknesses.

Selectively blocking the light using smart windows based on nanocolloids relies on the micro/nano‐sized aggregates with strong reflectance to the visible light and near‐infrared.^[^ ^55^ ^]^ For the proposed all‐climate management of temperature and light, smart windows need to block excessive solar radiation at high temperatures and prevent the dissipation of internal thermal radiation and the loss of plant supplementary light (**Figure** **3**a). Micro/nano‐sized aggregates generated by the heated p(NIPAM‐co‐DMAA) can effectively reflect the sunlight and reduce the *T_sol_* by 65.43–42.58%, achieving a maximum solar shading rate of 90.02% (Figure 3b; Figure S10a, Supporting Information). The heat‐induced thermochromism will decrease *T_PAR_* from highly transparent 91.18–85.28% to opaque 12.85–33.7% (Figure 3e). The *T_PAR_* increment of 15.4 and 4.1% in transparent and opaque states compared to the *T_sol_* illustrates the ability of smart windows to selectively filter the sunlight, which is caused by the absorption of PEG in 1000–2500 nm.^[^ ^25^ ^]^ Notably, the complete heat‐induced thermochromism requires a temperature change of ≈30 °C, meaning that continuous and mild regulation of the sunlight will be beneficial for efficient photosynthesis in plants rather than fast interrupting the light reaction of photosynthesis (Figure 3f; Figure S11a, Supporting Information). Being induced by low temperatures, micro/nano‐sized aggregates formed by the cooled p(AC‐co‐AM) enable the *T_sol_* to be below 5% (Figure S12, Supporting Information). Moreover, the PAR diffuse reflectance (*D_PAR_*) of 23.06–27.92% at low temperatures is a prerequisite for smart windows to improve the efficiency of plant supplementary lights in indoor farms (Figure 3h). The average diffuse reflectance and the average absorbance of 10.14–15.36% and 89.85–84.63% from 2.5 to 25 µm, respectively, allow partial interception of thermal radiation in indoor farms (Figure 3d; Figure S13, Supporting Information). Notably, with the reasonable design of the thermal response temperature, the strong infrared absorption of the smart window enables it to be quickly heated by sunlight, allowing for a rapid thermal response that prevents sunlight during hot daytime and allows sunlight to pass through during cold daytime. Overall, the strong IR absorption of the PEG–water matrix accelerates the temperature rise of the colloid‐based smart window, enabling a rapid thermochromic response without compromising thermal insulation. The relatively strong diffuse reflectance and absorbance of the light from 300 to 1000 nm by smart windows are beneficial for increasing the usage rate of light radiation generated by plant supplementary lights, allowing extra energy to be used to maintain indoor temperature. Compared to heat‐induced thermochromism, the complete cold‐induced thermochromism with >25% of *D_PAR_* changes only needs a temperature change of 15 °C, allowing for quick radiation regulation for indoor farms in the cold environment (Figure 3i; Figure S11b, Supporting Information). Furthermore, the regulation of *T_lum_* by p(AC‐co‐AM) is stronger than that by p(NIPAM‐co‐DMAA) (Figure S10b, Supporting Information), indicating that our smart window pays more attention to the radiation regulation degree and thermochromic speed in cold environments compared to hot environments. The intrinsic thickness dependence of window materials is very influential in optical performances.^[^ ^56^ ^]^ Increasing the thickness from 1 mm (91.01%) to 7 mm, the loss of *T_PAR_* in transparent smart windows is only 1.32% (Figure 3g; Figure S14a, Supporting Information). However, the increased thickness can significantly reduce *T_PAR_* (58.1 to 4.2%) and improve *D_PAR_* (13.53 to 30.29%) (Figure 3j; Figure S14b, Supporting Information).

The effects of salts on the optical performances of smart windows based on nanocolloids are shown in Figures S15 and S16 (Supporting Information). The unexpectedly decreased *T_PAR_* at the transparent state is caused by weak interaction between copolymers when the concentrations of salts are close to saturation.^[^ ^57^ ^]^ The steady *T_PAR_* of smart windows after the heat‐induced and cold‐induced thermochromism indicates that the type and concentration of salts weakly affect copolymers after phase changes. Therefore, regulating smart windows based on nanocolloids by salts requires the avoidance of the degradation of performance for light regulation due to salts with a high concentration.

The high economic efficiency of smart windows requires long‐lasting durability of the thermochromism.^[^ ^58^ ^]^ The freezing of the medium will break the hydrogen bonds between copolymers and water, rendering the hydrogen bonding‐dependent thermochromism ineffective.^[^ ^31^ ^]^ Hydrogen bonds between PEG and water can inhibit the crystallization of water molecules to delay the freezing of liquids.^[^ ^59^ ^]^ Figure S17a (Supporting Information) shows a decrease in the freezing point for nanocolloids from −0.93 to −44.1 °C by increased PEG proportion from 0 to 50%. The frost resistance of smart windows based on nanocolloids will maintain dynamic thermochromic performance, substantially improving the applicability in cold environments. In addition, the stability of optical performances is the core indicator of light regulation for smart windows.^[^ ^29^ ^]^ Figure S17b (Supporting Information) displays the retention of *T_PAR_*, *T_sol_*, and *D_PAR_* during 100 cycles between −20 and 80 °C. The fluctuation of *T_PAR_*, *T_sol_*, and *D_PAR_* within 2% indicates that smart windows based on nanocolloids have efficient reusability without causing the rapid degradation of optical performances. Although the precipitation of micro/nano‐sized aggregates after phase transition may lead to concerns in applications, particle aggregation appears not to be the bottleneck in this colloid system given the high viscosity (1000–3000 mPa·s) caused low sedimentation rate (Figure S18, Supporting Information), small sizes (<4000 nm), and the steric hindrance between particles caused by the high copolymer content (>17%).

### 2.3 Energy Saving and Temperature Regulation

**Figure 4:**
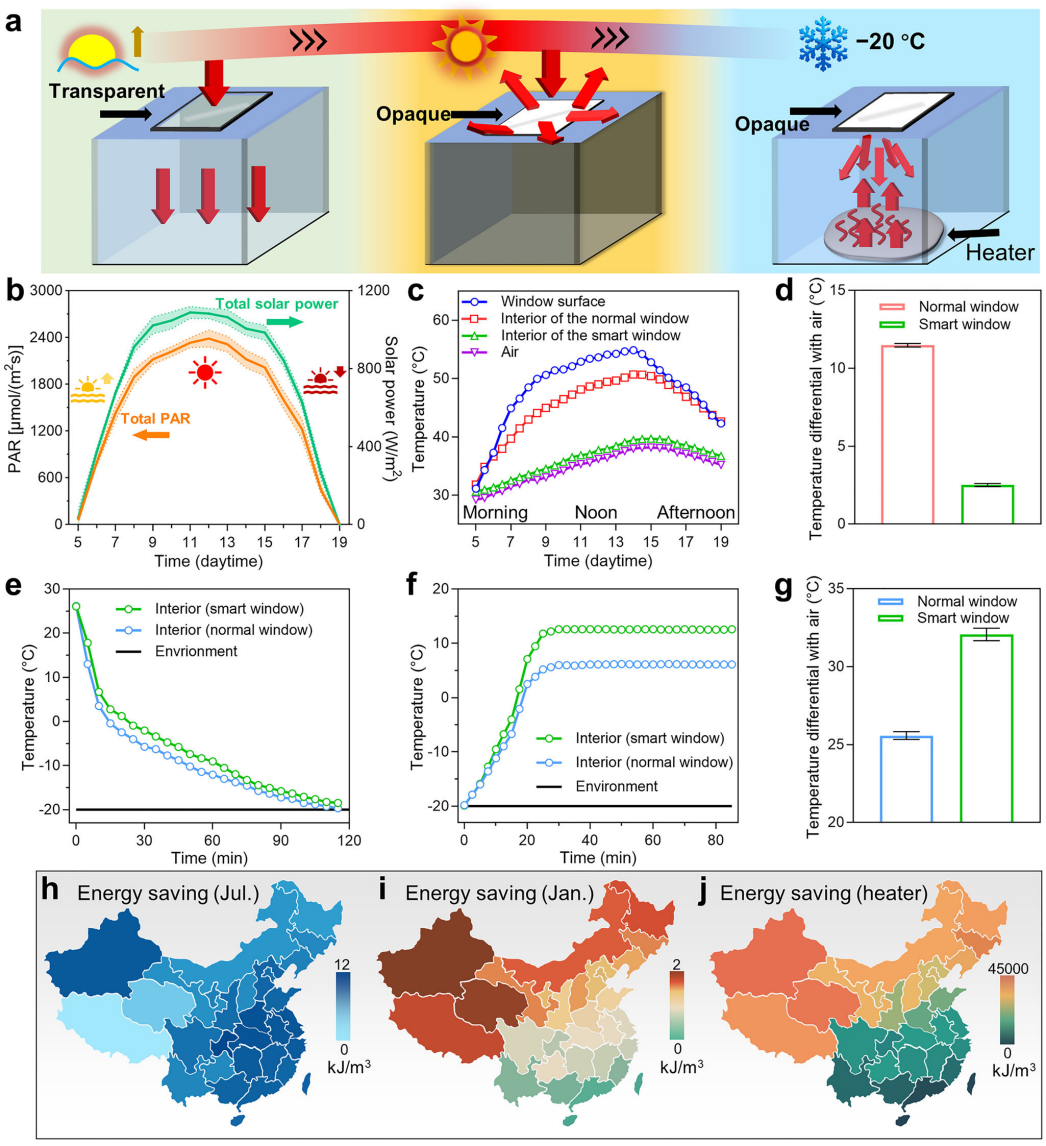
Energy saving by regulating thermal radiation of dual‐activation smart windows. a) Schematic illustration of the energy saving by the smart window in reflecting sunlight using heat‐induced thermochromism and circulating thermal radiation by cold‐induced thermochromism. b) The solar PAR and the solar power from 5:00 to 19:00. c) The window temperature and interior temperatures of the smart window‐covered box in the hot experiment. d) The average temperature differential between the air inside the boxes and the ambient air. e) Temperatures inside the boxes are moved into the environment of −20 °C during the cold simulation test. f) After turning on the heater (5 W), temperatures inside the boxes increase from −20 °C. g) Balance the temperatures inside the boxes after 30 min of heating. h,i) Assessments of energy saving in various provinces of China under natural conditions in July and January. j) The energy saving of the heater when reaching a constant temperature in January.

Our smart windows enable the mitigation of internal temperature fluctuations by diffusing thermal radiation inward or outward, thereby reducing the energy consumption of temperature control devices. Smart windows offer a transparent state in the morning to let sunlight enter and an opaque state at noon to reflect solar radiation for cooling the interior by heat‐induced thermochromism. The opaque smart window induced by the cold environment can strongly diffuse the thermal radiation from comparatively hotter objects inside the box, circulating the thermal radiation to warm the interior of the box (**Figure** **4**a; Figures S19 and S20, Supporting Information).^[^ ^30^ ^]^ Figure 4b records the all‐day solar PAR intensity and solar power, illustrating a test condition for intense sunlight exposure. In contrast to the normal window, the opaque smart window, when heated to an average temperature of 48.3 °C, can significantly reduce the average temperature inside the box from 44.2 to 35.2 °C (Figure 4c). The temperature differential of 9 °C caused by the smart window saves at least 11.61 kJ·m^−3^ of electrical energy consumption for the cooling (Figure 4d). Figure 4e details the cooling rate inside boxes covered with windows from a room condition to the −20 °C environment. Compared to the normal window, the smart window, which can raise the average temperature within the box by 2.3 °C, can conserve a minimum of 2.96 kJ·m^−3^ in energy required to maintain the internal warmth. Figure 4f records the internal temperature of boxes covered by windows in an environment of −20 °C after being heated by a heater (5W). Due to the thermochromism of colloids and the thermal radiation absorption of water and PEG, the diffuse reflection and strong absorption prevent the dissipation of thermal radiation emitted by the heater through the smart window. Therefore, the balanced temperature inside the box (12 °C) covered by the smart window is 6.4 °C higher than that of the control group (5.6 °C) (Figure 4g). Based on the energy loss calculation, the smart window is projected to conserve electrical energy by at least 2.21 kJ·(L·K)^−1^ over 24 h compared to the normal window, given a temperature differential of 32 °C between both boxes and the surroundings. The radiation regulation of bidirectional thermochromism will greatly benefit indoor farming by significantly saving electrical energy for temperature modulation, particularly in mainly electricity‐driven environment‐controlled agriculture. According to related energy saving performance, the energy saving caused by smart windows in different provinces of China under typical hot weather (July) and cold weather (January) is assessed through public weather data (Figure 4h–j). Under natural conditions, smart windows have a more significant cooling effect on southern provinces and a better insulation effect on northern provinces. In terms of the energy saving of heaters, there is a huge difference between the north and south, which is caused by the large temperature difference between the north and south of China during cold weather. These spatial energy saving assessments not only validate the practical potential of these smart windows across diverse climates but also establish a predictive model for tailoring material parameters to maximize energy efficiency in specific geographic regions.

### 2.4 Promoting Crop Growth Under Heat and Cold Stress

**Figure 5:**
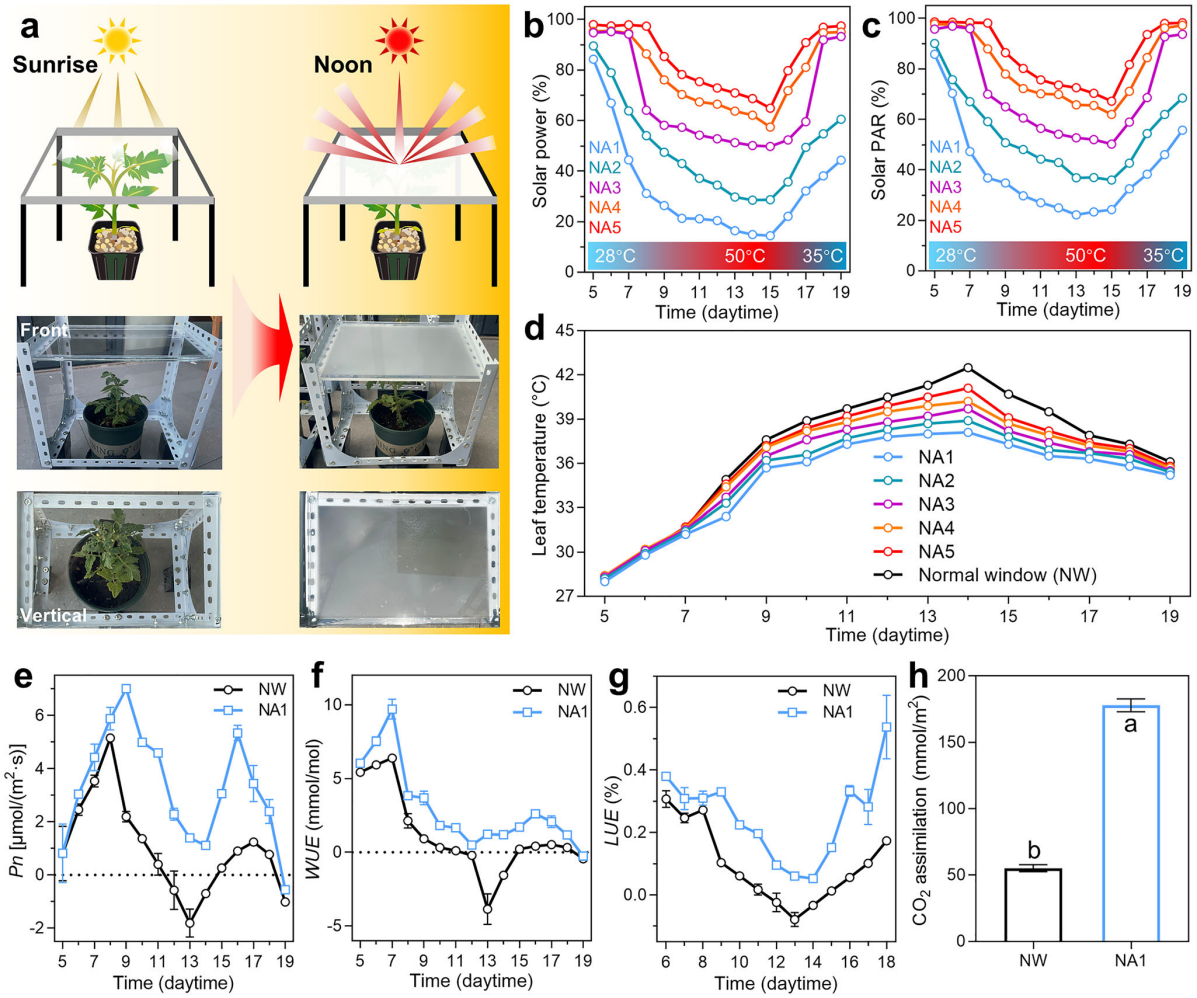
Promoting the cultivation of the non‐heat‐tolerant tomato seedlings under heat stress. a) The illustration depicts the 3‐mm‐thickness smart window turning transparent to opaque from sunrise to noon. The heat‐induced thermochromism of smart windows decreases the transmittance of (b) the solar power and c) the solar PAR, d) thus lowering the leaf temperature. e–h) Consequently, smart windows can significantly increase the *Pn*, *WUE*, and *LUE* of tomato seedlings under heat stress due to the cooling effect, thereby remarkably raising the CO_2_ assimilation. Detailed physiological indicators are shown in Figure S23 (Supporting Information). Different lowercase letters above the bars in (h) indicate significant differences (n = 10, *P* < 0.05).

Smart windows provide opportunities to enhance the all‐climate efficiency of sustainable agriculture, thereby diminishing the reliance on additional power. We have recreated two extreme planting environments to simulate planting conditions in regions characterized by large temperature differentials. **Figures** **5**a and S21 (Supporting Information) display the switching of the smart window from transparent in the morning to opaque at noon in cultivating tomato seedlings, achieving solar radiation regulation. As one of the most important global economic crops,^[^ ^60^ ^]^ heat stress is the main problem in the over‐summer planting of tomatoes.^[^ ^61^ ^]^ The non‐heat‐tolerant tomato seedlings generally require a suitable temperature lower than 35 °C to grow,^[^ ^62^ ^]^ meaning that the cultivation of tomato seedlings in Hangzhou (China, June, 120.2°E/30.3°N) is challenging due to the average daytime temperature reaching 35.4 °C (Figure S22, Supporting Information). Solar radiation enables the rapid heating of the window to diffuse the sunlight with heat‐induced thermochromism of smart windows, which have the highest temperature at 15:00. Smart windows with various heat‐induced temperatures will blur at different times. Figure 5b,c record transmittances of the solar power and PAR from sunrise to sunset. The NA1‐based smart window exhibits a continuous decrease in transmittance for solar power and solar PAR from 5:00 to 15:00, reducing to 14.46% and 23.35%, respectively. However, the NA5‐based smart window, which has an elevated thermochromic temperature and begins thermochromism between 8:00 and 9:00, only attains partial thermochromism at 15:00 with transmittances of 64.84% for solar power and 67.19% for solar PAR. Figure 5d depicts the daytime variation in leaf temperature distribution of tomato seedlings under different smart windows, detailing a reduction in the peak leaf temperature by 1.4–4.4 °C (14:00) relative to the normal window (NW). Figure 5e–g and Figure S23 (Supporting Information) record 6 physiological indicators of tomato leaves under NW and smart windows, including photosynthetic rate (*Pn*), transpiration rate (*Tr*), water use efficiency (*WUE*), stomatal conductance (*Gs*), intercellular CO_2_ concentration (*Ci*), and humidity change (*ΔRH*). The cooling effect offered by the smart window on tomato leaves mitigates the midday depression of photosynthesis caused by the heat stress, thereby maintaining stomata opening for continuous absorption of CO_2_ from the atmosphere.^[^ ^63^ ^]^ A reduction in temperature can mitigate leaf cell respiration and enhance the activity of photosynthetic enzymes, consequently leading to a positive *Pn* in tomato seedlings during the daytime. Under the NW, overheated leaves close their stomata, resulting in respiration consumption exceeding photosynthetic assimilation accumulation.^[^ ^64^ ^]^ Furthermore, the enhanced *WUE* of leaves indicates that the reduction in transpiration intensity due to smart windows is beneficial for conserving water resources in agriculture. Although smart windows reduce the solar PAR transmittance during the daytime, they overall improve the light use efficiency (*LUE*) of tomato seedlings, as illustrated in Figure S24a (Supporting Information). Therefore, the temperature regulation provided by smart windows, which resists heat stress, can significantly promote the growth of tomato seedlings and increase CO_2_ assimilation by up to 222.19%, as depicted in Figure 5h and Figure S19b (Supporting Information). In brief, smart windows can effectively alleviate the heat stress of indoor crops, enabling them to overcome the growth bottleneck imposed by the deterioration of physiological indicators from solar heating.

**Figure 6:**
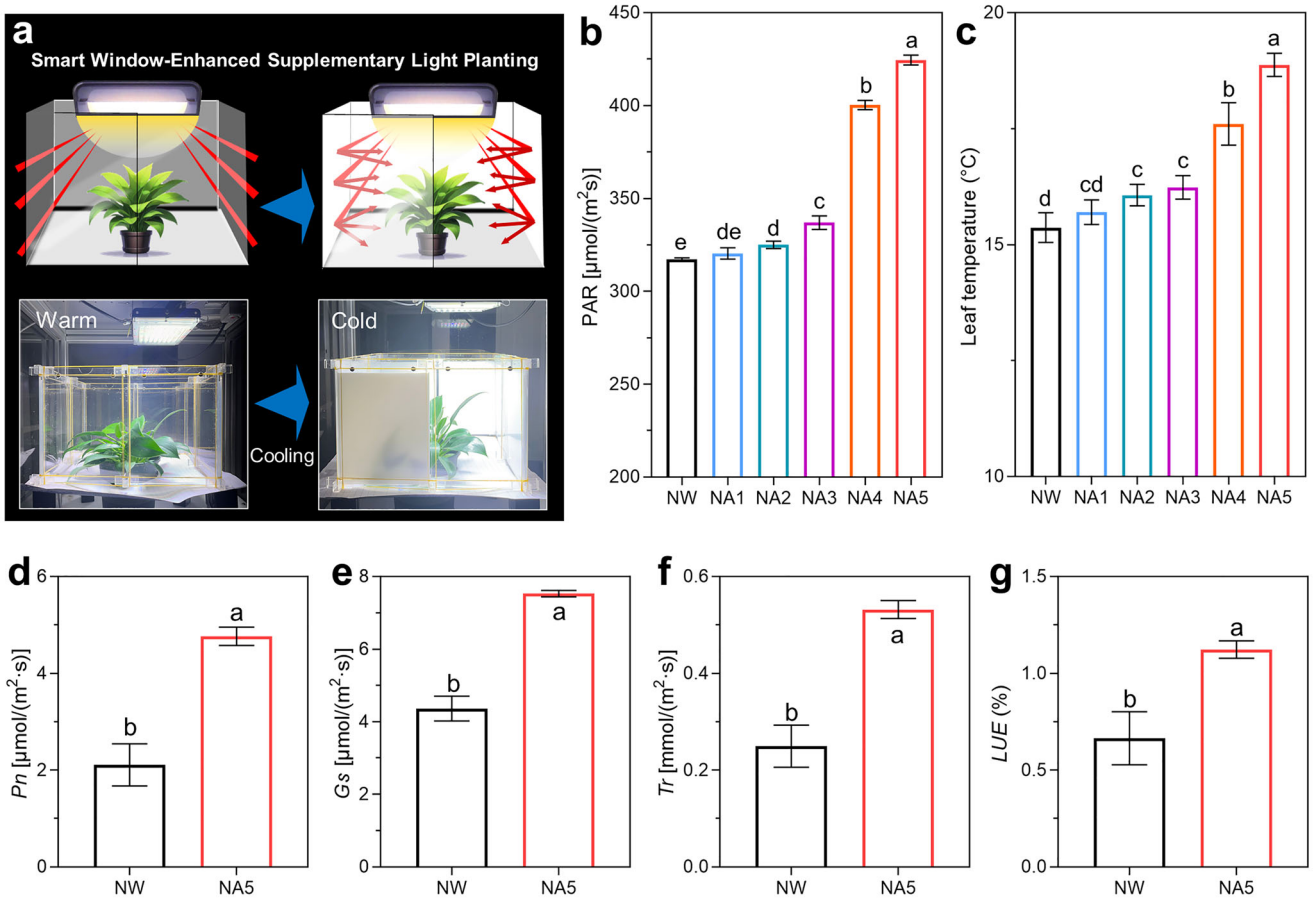
Promoting the cultivation of the non‐cold‐tolerant *Epipremnum aureum*. a) The illustration describes the 3‐mm‐thickness smart window turning transparent to opaque (from warm to cold environments). The cold‐thermochromic smart window reflects (b) the PAR of the plant supplementary lamp and heats (c) the leaves by reflecting the thermal radiation. d–g) The enhanced PAR and warmed leaves by the smart window can prevent stomata from closing under cold stress and maintain the activity of respiration and photosynthetic enzymes, thereby increasing the *LUE* and *Pn*. Detailed physiological indicators are shown in Figures S27 and S28 (Supporting Information). Different lowercase letters above the bars in (b–g) indicate significant differences (n = 10, *P* < 0.05).

Planting by supplementary light in indoor agriculture is now extensively implemented worldwide, particularly in greenhouses and vertical farms that depend on such lighting to facilitate photosynthesis.^[^ ^65^ ^]^ **Figures** **6**a and S25 (Supporting Information) show the reversible transition of the smart window between transparency and opacity in response to cold conditions, thereby enabling a controlled modulation of thermal radiation and PAR emitted by the supplementary lamp. *Epipremnum aureum*, commonly known as Golden Pothos, is a species that thrives in warm climates and is extensively cultivated indoors globally.^[^ ^66^ ^]^ Setting the ambient temperature to 10 °C, the lower limit for *Epipremnum aureum* growth, Figure 6b depicts a substantial boost in PAR intensity near the leaves, peaking at 33.9%, caused by the cold‐induced opacity of the NA5‐based smart window. Conversely, transparent NA1–NA3‐based smart windows featuring a UCST below 10 °C fail to achieve a notable increase in PAR. The efficacy of supplementary light planting is inherently distance‐dependent.^[^ ^67^ ^]^ With increasing distance between leaves and the light source, the expanded reflective area of opaque smart windows correspondingly boosts the reflected PAR received by the leaves. This results in a distance‐dependent enhancement of PAR, with an increase ranging from 4.9% to 35.7% as the distance extends from 10 cm to 40 cm, as shown in Figure S26 (Supporting Information). The opaque NA5‐based smart window enables the effective reflection of thermal radiation emitted by the PAR lamp, resulting in a 22.8% increase in the leaf temperature (Figure 6c). Figure 6d demonstrates a remarkable 126.07% increase in *Pn* of leaves, attributable to the NA5‐based smart window. The cold stress will induce stomatal closure in the leaf epidermis and decrease the activity of photosynthetic enzymes, thus diminishing the uptake of CO_2_ and concurrently reducing the intensity of light‐dependent reactions.^[^ ^68^ ^]^ Consequently, the radiation regulation of smart windows, which warms the leaves and enhances PAR, can stimulate stomatal opening and elevate respiration and enzyme activity, thus boosting CO_2_ uptake and intensifying the light‐driven reactions, as depicted in Figure 6e,f. Solving cold stress by the NA5‐based smart window enables a significant 68.8% increase in the *LUE* of leaf cells (Figure 6g). In short, smart windows effectively alleviate the cold stress for indoor crops and enhance the energy utilization efficiency of supplementary lighting systems, which are achieved by providing thermal preservation and improving the efficacy of supplementary lighting.

## 3 Conclusion

We demonstrate a bidirectionally and extensively thermochromic nanocolloid‐based optical switching strategy for smart windows designed to improve the efficiency of sustainable agriculture. The smart window adopts two individual thermo‐responsive copolymers, influenced by the Hofmeister effect and the cononsolvency of PEG and water, to achieve dual thermochromism across a broad temperature range of heat‐responsive 27–85 °C and cold‐responsive −9–36 °C. In addition to the transparent *T_PAR_* reaching 91.18%, the smart window also effectively modulates indoor temperatures by managing solar and indoor thermal radiation, realizing energy savings of 11.61 kJ·m^−3^ in a hot environment and 2.96 kJ·m^−3^ in a cold environment. The management of PAR and temperature by smart windows significantly boosts the photosynthetic intensity of non‐heat‐tolerant tomato seedlings and non‐cold‐tolerant *Epipremnum aureum* by 222.19% and 126.07% under heat and cold stress, respectively. The exceptional environmental stability and reusability underscore the practicality of smart windows. Ultimately, our nanocolloid‐based smart window, characterized by its ease of production, customization, and replaceability, showcases great potential in conserving power within all‐climate electro‐agriculture, promoting the efficiency of off‐season farming and supplementary light cultivation, and securing food. This pioneering study, utilizing dynamic and complex hydrogen bonding regulation between dispersed phases and the medium, advances the application of smart windows in promoting the decarbonization of sustainable agriculture.

## 4 Experimental Section

### Materials

*N*‐isopropylacrylamide (NIPAM, >99%), *N,N*‐dimethylacrylamide (DMAA, >99%), acrylic acid (AC, >99%), and acrylamide (AM, >99%) were purchased from Yuanye Bio‐Tech. (Shanghai, China). Polyethylene glycol (PEG400, >99%), sodium salts (AR), potassium salts (AR), ammonium persulfate (APS, AR), and *N*,*N*,*N″*,*N″*‐tetramethylethylenediamine (TED, >99%) were obtained from MACKLIN (Shanghai, China). Ultra‐pure water (18.2 MΩ·cm) was generated by the laboratory water purification system (SU‐20UV, HyperPureX, China).

### Preparation of Nanocolloids and Smart Windows

PEG and ultra‐pure water were mixed in a ratio of 0.8:1 (vol./vol.) to prepare the continuous medium for organo‐hydrocolloids. For preparing heat‐responsive P(NIPAM‐co‐DMAA) nanocolloids, NIPAM (800 mg), DMAA (500–540 mg), and APS (14 mg) were completely dissolved in the medium (10 mL) at 25 °C. With 600 rpm of the continuous mechanical stir and 25 °C of environment, TED (14 mL) was quickly added into the solution to start free radical polymerization between NIPAM and DMAA, which needed 2 h to end the reaction. For preparing cold‐responsive P(AC‐co‐AM) nanocolloids, AC (300 mg), AM (500–1300 mg), and APS (20 mg) were completely dissolved in the medium (10 mL) at 45 °C. With 900 rpm of the continuous mechanical stir and 45 °C of environment, TED (25 mL) was quickly added into the solution to start free radical polymerization between AC and AM, which needed 4 h to end the reaction. The dual‐directional and extensively thermochromic nanocolloids (NA1–NA5) were prepared by mixing volume‐equal liquids containing P(NIPAM‐co‐DMAA) and P(AC‐co‐AM) using a 600 rpm mechanical stir for 1 h (20 °C). Detailed synthetic formulas of organo‐hydrocolloids are listed in Table S1 (Supporting Information). Smart windows were prepared using a facile method that directly injected liquid nanocolloids into normal insulating glass windows using a syringe.

### Salt Effects

As for nanocolloids with sodium and potassium salts, salts of different concentrations (0.2–1 mol L^−1^) were added soon after ending the free radical polymerization, along with 600 rpm of mechanical stir for 1 h before mixing P(NIPAM‐co‐DMAA) and P(AC‐co‐AM). For preparing P(NIPAM‐co‐DMAA), the doses of NIPAM and DMAA are 800 and 500 mg, respectively. For preparing P(AC‐co‐AM), the doses of AC and AM are 300 and 500 mg, respectively.

### Differential Scanning Calorimetry (DSC)

The thermochromism‐related temperature was measured by DSC (DSC1 Pro, METTLER, Switzerland). The DSC testing cycle started from 25, 30, 35, 40, and 45 °C for NA1‐NA5, respectively, which then rose to 95 °C, dropped to −20 °C, and rose to the initial temperature. The testing rate was set as 5 °C min^−1^. Nitrogen (>99.9%, 30 mL min^−1^) was offered as the atmospheric condition. Around 20 mg liquid samples were added to the aluminum pan (40 µL) for measurement. *T_onset_* represented the initial temperature at which thermal effects occurred, while *T_c_* represented the temperature of peaks in DSC.

### Optical Performances of Smart Windows

The transmittance and reflection spectra between 280–2500 nm of nanocolloids in the quartz colorimetric dish with different optical paths (1, 3, 5, and 7 mm) were scanned by the in situ (temperature variable) ultraviolet spectrometer (UV‐3600 iplus, Shimadzu, Japan). The reflection and transmittance spectra in the range of 2.5–25 µm were scanned by Nicolet iS50 (Thermo Scientific, USA), that loaded with a temperature control accessory. The transmittance of photosynthetically active radiation (*T_PAR_*), luminosity function (*T_lum_*), and solar light (*T_sol_*), and the diffuse reflectance of photosynthetically active radiation (*D_PAR_*) were calculated by Equations (1), (2), (3), (4):^[^ ^25^ ^]^

(1) $$T_{\mathrm{PAR}}=\frac{\int \varphi_{\mathrm{PAR}}T\left(\lambda \right)d\lambda }{\int \varphi_{\mathrm{PAR}}d\lambda }$$

(2) $$T_{\mathrm{lum}}=\frac{\int \varphi_{\mathrm{lum}}T\left(\lambda \right)d\lambda }{\int \varphi_{\mathrm{lum}}d\lambda }$$

(3) $$T_{sol}=\frac{\int \varphi_{sol}T\left(\lambda \right)d\lambda }{\int \varphi_{sol}d\lambda }$$

(4) $$D_{\mathrm{PAR}}=\frac{\int \varphi_{\mathrm{PAR}}D\left(\lambda \right)d\lambda }{\int \varphi_{\mathrm{PAR}}d\lambda }$$

where *φ_PAR_*, *φ_lum_*, and *φ_sol_* represent the McCree curve (350–750 nm, Figure S29a, Supporting Information),^[^ ^69^ ^]^ the standard luminous efficiency function (360–780 nm, Figure S29b, Supporting Information), and the solar spectrum (250–2500 nm, Figure S29c, Supporting Information), respectively. *T(λ)* and *D(λ)* are the transmittance and diffuse reflectance, respectively.

### Size Measurement of Nanocolloids

The particle size of colloids during thermochromism was measured by Zetasizer Nano (ZS90, Malvern, UK), which loaded a temperature control accessory. Organo‐hydrocolloids were diluted with 9 times the volume of the same medium before testing. For testing the particle size during both heat‐induced and cold‐induced thermochromism, the testing temperature started from 25, 30, 35, 40, and 45 °C for NA1–NA5, respectively. After an equilibrium time of 2 min, the liquid sample (≈1 mL) in the sample cell (DTS0012) was tested three times.

### Density Functional Theory (DFT) Calculation

The solvation free energy of NIPAM‐DMAA and AC‐AM in the neat water was calculated using by software Gaussian 16.^[^ ^70^ ^]^ The binding ability between models and water molecules at 253, 293, and 368 K was calculated by the implicit solvation model, avoiding the influence of the complexity of multiple binding conformations between polymers and water. The density functional B3LYP^[^ ^71^ ^]^ with the D3BJ dispersion correction^[^ ^72^ ^]^ was used for calculations. The geometry optimization was performed with a basis set of the 6‐311G+(d,p).^[^ ^73^, ^74^ ^]^ The single‐point energy and thermodynamic calculation were performed under M06‐2X/6‐311G+(d,p) with the SMD solvent model of water.^[^ ^75^ ^]^

### Simulated Tests of Dual‐Directional Temperature Regulation

The insulating glass window was used as the control group in simulated tests. In the high‐temperature simulation, the NA1‐based smart window was used to cover the surface of sealed foam boxes that contained temperature and optical detection probes. These boxes were placed in a location that could be exposed to sunlight throughout the day (June, Hangzhou, China). The transmittance of solar PAR and the power of the solar light were tested by a handheld photosynthetic radiometer (SM206‐PAR, Sanpometer, China) and a handheld solar power meter (SM206‐SOLAR, Sanpometer, China). According to Equation 5, the energy saving in the high‐temperature simulation was calculated. In the low‐temperature simulation, a refrigeration device (−20–20 °C) was used to simulate a cold environment. Firstly, boxes, which were covered with the NA5‐based smart window and the control window, were quickly transferred from 25 °C to the refrigeration device at −20 °C, in which the temperature was continuously monitored. The energy saving was also calculated using Equation 4. Then, plate heaters with 5 W power were added to the boxes, respectively. In the refrigeration device at −20 °C, heaters were turned on to heat the air inside the boxes. With heaters, the energy‐saving efficiency of the smart window was calculated by Equations (5), (6), (7), (8):^[^ ^25^, ^29^ ^]^

(5) $$\mathrm{Energy}\;\mathrm{saving}=\pm \;\left(\bar{T_{\mathrm{control}}}-\bar{T_{\mathrm{test}}}\right)\rho_{\mathrm{air}}C_{\mathrm{air}}$$

where $\bar{T_{control}}$ and $\bar{T_{test}}$ are the average temperature of the control group and test group, respectively, *ρ_air_* is the density of air (1.29 kg m^−3^), and *C_air_* is the specific heat capacity of air (1.004 kJ kg^−1^ K^−1^).

(6) $$\Delta Q=W-\bar{\Delta T}\left(V\rho_{\mathrm{air}}C_{\mathrm{air}}\right)-\frac{\bar{\Delta T}}{2}\left(M_{b}C_{b}+M_{w}C_{w}\right)$$

where *ΔQ* is the thermal energy loss of boxes, *W* is electric energy consumption, $\bar{\Delta T}$ is the average temperature differential inside and outside boxes, and *V* is the volume of the box. *M_b_* and *M_w_* are the mass of the box and window, respectively. *C_b_* and *C_w_* are the specific heat capacities of the box (1.5 kJ kg^−1^ K^−1^) and window (0.75 kJ kg^−1^ K^−1^), respectively.

(7) $$\Delta E=\frac{1}{V}\left(\frac{\Delta Q_{\mathrm{control}}}{\bar{\Delta T_{\mathrm{control}}}}-\frac{\Delta Q_{\mathrm{test}}}{\bar{\Delta T_{\mathrm{test}}}}\right)$$

where *ΔE* (J·L^−1^·K^−1^) is the difference in thermal energy loss of test and control groups, ΔQ_control_ and ΔQ_test_ are the thermal energy loss in the control and test groups, respectively, and $\bar{\Delta T_{\mathrm{control}}}$ and $\bar{\Delta T_{\mathrm{test}}}$ are the average temperature differentials inside and outside the control and test boxes, respectively.

(8) $$\mathrm{Energy}\;\mathrm{saving}\;\mathrm{efficiency}=\Delta E\cdot V\cdot \bar{\Delta T}$$

The assessment of energy saving in various provinces of China was based on public weather data from the China Meteorological Administration.

### Simulated Indoor Farming with Smart Windows

In the simulated test of the hot environment, common potted tomato seedlings (*Solanum lycopersicum L*.) were adopted as the representative non‐heat‐tolerant crops, which are not heat‐resistant (<35 °C). Smart windows were fixed on shelves to simulate the glass room, which was exposed to sunlight all day. Potted tomato seedlings were placed under smart windows for cultivation and thoroughly watered after every sunset. From sunrise to sunset, the physiological status of leaves (photosynthetic rate (*Pn*), transpiration rate (*Tr*), water use efficiency (*WUE*), stomatal conductance (*Gs*), intercellular CO_2_ concentration (*Ci*), and humidity change (*ΔRH*)) was tested by the photosynthesis analyzer (HED‐GH20, HORDE ELECTRIC, China), which loaded a probe equipped with a CO_2_ detector, a humidity detector, a light intensity detector, and a leaf chamber of 3 m^2^. In the simulated test of the cold environment, a 1 m^2^ transparent box with the temperature control module was used to keep the ambient temperature ≈10 °C and was placed in a dark room. The tested plant was *Epipremnum aureum f. “Jade”*is an indoor cultivated crop suitable for growing in warm environments and widely grown in greenhouses around the world. Smart windows were used to surround potted *Epipremnum aureum*, which was illuminated by a light source (100 W, full wavelength of PAR) for assisted growth. Potted *Epipremnum aureum* received 4 h of supplementary light every day and was watered regularly. The physiological status of leaves was tested using the photosynthesis analyzer after continuous exposure to the light source for 1 h. The light use efficiency (*LUE*) and CO_2_ assimilation were calculated by Equations 9 and 10:

(9) $$LUE=\frac{Pn}{PAR}$$

(10) $$CO_{2}\;\mathrm{assimilation}=\int \mathrm{Pn}\left(t\right)\mathrm{dt}$$

where *Pn(t)* is the photosynthetic rate at time *t*. A window group contains ten potted crops.

### FTIR Spectroscopy

The FTIR spectra were measured by Nicolet iS10 (Thermo Scientific, USA). P(NIPAM‐co‐DMAA) and P(AC‐co‐AM) were squeezed to remove liquids when heated or cooled to opaque states, respectively. After removing liquids and dehydration, solid samples were tested using an ATR attachment of the spectrometer. The testing range was from 400 to 4000 cm^−1^ with a resolution of 2 cm^−1^.

### Cycling Tests of High and Low Temperatures

The cycling tests of changing temperature were conducted using a temperature chamber (BPH‐060A, Shanghai Yiheng, China) that could regulate the temperature by programmed approaches. NA1 nanocolloids were placed in the chamber with a cyclically adjustable temperature between −20 to 80 °C. The transmittance and diffuse reflectance of NA1 nanocolloids were tested every 10 temperature cycles. *T_PAR_*, *T_sol_*, and *D_PAR_* were calculated to assess changes in optical performances.

### Rheology

The viscosity of nanocolloids was measured by the MCR302 rheometer (Anton Paar, Austria). NA1–NA5 were collected and placed on the testing plate at 25 °C using a plate probe with a 60‐mm diameter to confine liquid samples in a 1‐mm‐thickness gap for measurement. The shear rate increased from 0.01 to 100 s^−1^; every increased order of magnitude had 10 measuring points.

### Statistical Analysis

Data were presented as the mean ± standard deviation. Each statistical analysis included at least five samples (n ≥ 5). One‐way ANOVA and Tukey's test were used to assess significant differences (*P* < 0.05). OriginLab (9.0) was used for data analysis and graphing.

## Conflict of Interest

The authors declare no conflict of interest.

## Supporting information



Supporting Information

## Data Availability

The data that support the findings of this study are available from the corresponding author upon reasonable request.
