# Supplementary material for: Bidirectionally Thermochromic Nanocolloid System for on‐Demand Optical Switching and Agricultural Energy Management
Source: Adv Sci (Weinh). 2025 Nov 12;13(12):e19759. doi: 10.1002/advs.202519759 (PMC12948220; doi:10.1002/advs.202519759)
Supplement: Supplementary file 1 — Supporting Information [file ADVS-13-e19759-s001.docx]

**Supporting Information**

**Bidirectionally Thermochromic Nanocolloid System for On-Demand Optical Switching and Agricultural Energy Management**

Qinbo Jiang^1^, Jiayi Li^1^, and Hui Zhang^1,*^

^1^ College of Biosystems Engineering and Food Science, Zhejiang University, Hangzhou, 310058, China.

* Corresponding author: [hubert0513@zju.edu.cn](mailto:hubert0513@zju.edu.cn)

Table S1. The synthetic formula and *T_c_* of p(NIPAM-co-DMAA) and p(AC-co-AM) for heat-induced and cold-induced thermochromism, respectively. NA1–NA5 mean the final organo-hydrocolloids after mixing liquids containing p(NIPAM-co-DMAA) and p(AC-co-AM).

| Groups | P(NIPAM-co-DMAA) | | | | |  | P(AC-co-AM) | | | | |
| --- | --- | --- | --- | --- | --- | --- | --- | --- | --- | --- | --- |
|  | NIPAM  (mg) | DMAA  (mg) | APS  (mg) | TED  (mL) | *T_c_* (°C) |  | AC (mg) | AM  (mg) | APS  (mg) | TED  (mL) | *T_c_* (°C) |
| NA1 | 800 | 500 | 14 | 14 | 67.36 ± 0.90^e^ |  | 300 | 500 | 20 | 25 | −12.5 ± 0.25^e^ |
| NA2 | 800 | 510 | 14 | 14 | 72.43 ± 0.15^d^ |  | 300 | 700 | 20 | 25 | −5.13 ± 0.25^d^ |
| NA3 | 800 | 520 | 14 | 14 | 79.30 ± 0.39^c^ |  | 300 | 900 | 20 | 25 | 2.43 ± 0.15^c^ |
| NA4 | 800 | 530 | 14 | 14 | 87.93 ± 1.02^b^ |  | 300 | 1100 | 20 | 25 | 10.60 ± 0.30^b^ |
| NA5 | 800 | 540 | 14 | 14 | 95.06 ± 1.23^a^ |  | 300 | 1300 | 20 | 25 | 17.73 ± 0.40^a^ |

Different letters mean the significant difference (*P* < 0.05).

Table S2. *T_onset_* and *T_c_* of NA1–NA5 organo-hydrocolloids.

| Groups | Heat-induced thermochromism | |  | Cold-induced thermochromism | |
| --- | --- | --- | --- | --- | --- |
|  | *T_onset_* (°C) | *T_c_* (°C) |  | *T_onset_* (°C) | *T_c_* (°C) |
| NA1 | 27.13 ± 1.18^e^ | 38.53 ± 0.66^e^ |  | −5.76 ± 0.51^e^ | −9.83 ± 0.35^e^ |
| NA2 | 33.80 ± 1.67^d^ | 43.10 ± 0.72^d^ |  | 0.43 ± 0.20^d^ | −4.70 ± 0.45^d^ |
| NA3 | 39.73 ± 1.40^c^ | 59.73 ± 1.60^c^ |  | 10.43 ± 0.70^c^ | 5.03 ± 0.20^c^ |
| NA4 | 45.70 ± 0.45^b^ | 68.40 ± 0.65^b^ |  | 24.36 ± 0.66^b^ | 13.13 ± 1.00^b^ |
| NA5 | 50.10 ± 0.88^a^ | 85.16 ± 1.17^a^ |  | 36.83 ± 0.35^a^ | 18.03 ± 0.97^a^ |

Different letters mean the significant difference (*P* < 0.05).

Table S3. Effects of four sodium salts on heat-induced *T_onset_* and *T_c_* of organo-hydrocolloids.

| Concentration (mol/L) | NaCl | |  | NaI | |  | NaBr | |  | Na_2_SO_4_ | |
| --- | --- | --- | --- | --- | --- | --- | --- | --- | --- | --- | --- |
|  | *T_onset_* (°C) | *T_c_* (°C) |  | *T_onset_* (°C) | *T_c_* (°C) |  | *T_onset_* (°C) | *T_c_* (°C) |  | *T_onset_* (°C) | *T_c_* (°C) |
| 0 | 54.4 ± 0.2^a^ | 66.7 ± 0.9^a^ |  | 54.4 ± 0.2^f^ | 66.7 ± 0.9^f^ |  | 54.4 ± 0.2^a^ | 66.7 ± 0.9^a^ |  | 54.4 ± 0.2^a^ | 66.7 ± 0.9^a^ |
| 0.2 | 40.1 ± 0.3^b^ | 54.7 ± 0.6^b^ |  | 60.1 ± 0.2^e^ | 69.1 ± 0.4^e^ |  | 48.5 ± 0.2^b^ | 56.5 ± 0.1^b^ |  | 42.1 ± 0.5^b^ | 47.6 ± 0.6^b^ |
| 0.4 | 34.5 ± 0.4^c^ | 43.3 ± 0.7^c^ |  | 65.1 ± 0.3^d^ | 73.8 ± 0.4^d^ |  | 44.3 ± 0.4^c^ | 47.6 ± 0.1^c^ |  | \ | \ |
| 0.6 | 18.1 ± 0.5^d^ | 34.6 ± 0.6^d^ |  | 69.4 ± 0.2^c^ | 77.1 ± 0.3^c^ |  | 40.1 ± 0.4^d^ | 44.7 ± 0.2^d^ |  | \ | \ |
| 0.8 | 16.2 ± 0.3^e^ | 23.3 ± 0.5^e^ |  | 72.2 ± 0.3^b^ | 79.2 ± 0.3^b^ |  | 37.4 ± 0.4^e^ | 40.2 ± 0.2^e^ |  | \ | \ |
| 1.0 | 6.2 ± 0.2^f^ | 12.1 ± 0.4^f^ |  | 75.7 ± 0.3^a^ | 80.8 ± 0.3^a^ |  | 34.1 ± 0.3^f^ | 36.1 ± 0.2^f^ |  | \ | \ |

Different letters mean the significant difference (*P* < 0.05).

Table S4. Effects of four potassium salts on heat-induced *T_onset_* and *T_c_* of organo-hydrocolloids.

| Concentration (mol/L) | KCl | |  | KI | |  | KBr | |  | K_2_SO_4_ | |
| --- | --- | --- | --- | --- | --- | --- | --- | --- | --- | --- | --- |
|  | *T_onset_* (°C) | *T_c_* (°C) |  | *T_onset_* (°C) | *T_c_* (°C) |  | *T_onset_* (°C) | *T_c_* (°C) |  | *T_onset_* (°C) | *T_c_* (°C) |
| 0 | 54.4 ± 0.2^a^ | 66.7 ± 0.9^a^ |  | 54.4 ± 0.2^f^ | 66.7 ± 0.9^f^ |  | 54.4 ± 0.2^a^ | 66.7 ± 0.9^a^ |  | 54.4 ± 0.2^a^ | 66.7 ± 0.9^a^ |
| 0.2 | 40.1 ± 0.3^b^ | 54.7 ± 0.4^b^ |  | 59.8 ± 0.3^e^ | 67.1 ± 0.4^e^ |  | 45.5 ± 0.1^b^ | 55.1 ± 0.4^b^ |  | 8.5 ± 0.3^b^ | 19.2 ± 0.6^b^ |
| 0.4 | 34.4 ± 0.4^c^ | 43.1 ± 0.4^c^ |  | 62.2 ± 0.4^d^ | 68.4 ± 0.3^d^ |  | 40.1 ± 0.3^c^ | 44.3 ± 0.5^c^ |  | \ | \ |
| 0.6 | 26.1 ± 0.5^d^ | 34.2 ± 0.2^d^ |  | 66.1 ± 0.5^c^ | 70.4 ± 0.3^c^ |  | 36.5 ± 0.3^d^ | 39.2 ± 0.4^d^ |  | \ | \ |
| 0.8 | 5.1 ± 0.4^e^ | 16.2 ± 0.2^e^ |  | 70.3 ± 0.3^b^ | 73.4 ± 0.2^b^ |  | 29.6 ± 0.2^e^ | 34.6 ± 0.5^e^ |  | \ | \ |
| 1.0 | −3.9 ± 0.2^f^ | 6.1 ± 0.4^f^ |  | 73.6 ± 0.2^a^ | 75.7 ± 0.4^a^ |  | 22.8 ± 0.3^f^ | 28.2 ± 0.4^f^ |  | \ | \ |

Different letters mean the significant difference (*P* < 0.05).

Table S5. Cold-induced *T_onset_* and *T_c_* of organo-hydrocolloids with adding four sodium salts.

| Concentration (mol/L) | NaCl | |  | NaI | |  | NaBr | |  | Na_2_SO_4_ | |
| --- | --- | --- | --- | --- | --- | --- | --- | --- | --- | --- | --- |
|  | *T_onset_* (°C) | *T_c_* (°C) |  | *T_onset_* (°C) | *T_c_* (°C) |  | *T_onset_* (°C) | *T_c_* (°C) |  | *T_onset_* (°C) | *T_c_* (°C) |
| 0 | −8.66 ± 0.15^d^ | −10.56 ± 0.30^d^ |  | −8.66 ± 0.15^f^ | −10.56 ± 0.30^e^ |  | −8.66 ± 0.15^f^ | −10.56 ± 0.30^f^ |  | −8.66 ± 0.15^a^ | −10.56 ± 0.30^a^ |
| 0.2 | −2.36 ± 0.30^a^ | −4.23 ± 0.15^a^ |  | 8.366 ± 0.11^e^ | 3.33 ± 0.30^d^ |  | −1.63 ± 0.15^e^ | −3.43 ± 0.25^e^ |  | −11.80 ± 0.10^b^ | −13.90 ± 0.17^b^ |
| 0.4 | −3.20 ± 0.10^b^ | −5.83 ± 0.05^b^ |  | 10.23 ± 0.15^d^ | 8.13 ± 0.25^c^ |  | 2.20 ± 0.26^d^ | 0.53 ± 0.15^d^ |  | \ | \ |
| 0.6 | −5.46 ± 0.25^c^ | −7.96 ± 0.30^c^ |  | 15.86 ± 0.20^c^ | 13.06 ± 0.15^b^ |  | 4.83 ± 0.11^c^ | 2.20 ± 0.30^c^ |  | \ | \ |
| 0.8 | −8.40 ± 0.09^d^ | −10.80 ± 0.19^d^ |  | 17.73 ± 0.15^b^ | 14.36 ± 0.15^a^ |  | 5.36 ± 0.15^b^ | 3.13 ± 0.20^b^ |  | \ | \ |
| 1.0 | −9.36 ± 0.25^e^ | −12.56 ± 0.41^e^ |  | 19.43 ± 0.20^a^ | 14.66 ± 0.15^a^ |  | 6.70 ± 0.10^a^ | 4.06 ± 0.15^a^ |  | \ | \ |

Different letters mean the significant difference (*P* < 0.05).

Table S6. Cold-induced *T_onset_* and *T_c_* of organo-hydrocolloids with adding four potassium salts.

| Concentration (mol/L) | KCl | |  | KI | |  | KBr | |  | K_2_SO_4_ | |
| --- | --- | --- | --- | --- | --- | --- | --- | --- | --- | --- | --- |
|  | *T_onset_* (°C) | *T_c_* (°C) |  | *T_onset_* (°C) | *T_c_* (°C) |  | *T_onset_* (°C) | *T_c_* (°C) |  | *T_onset_* (°C) | *T_c_* (°C) |
| 0 | −8.66 ± 0.15^d^ | −10.56 ± 0.30^d^ |  | −8.66 ± 0.15^f^ | −10.56 ± 0.30^f^ |  | −8.66 ± 0.15^f^ | −10.56 ± 0.30^f^ |  | −8.66 ± 0.15^a^ | −10.56 ± 0.30^a^ |
| 0.2 | −2.13 ± 0.05^a^ | −4.50 ± 0.10^a^ |  | 8.26 ± 0.15^e^ | 2.63 ± 0.15^e^ |  | 1.10 ± 0.20^e^ | −3.30 ± 0.26^e^ |  | −11.76 ± 0.11^b^ | −13.96 ± 0.15^b^ |
| 0.4 | −3.60 ± 0.09^b^ | −5.40 ± 0.09^b^ |  | 12.63 ± 0.15^d^ | 8.10 ± 0.17^d^ |  | 3.56 ± 0.25^d^ | −0.10 ± 0.30^d^ |  | \ | \ |
| 0.6 | −6.86 ± 0.05^c^ | −8.96 ± 0.11^c^ |  | 17.06 ± 0.15^c^ | 12.50 ± 0.09^c^ |  | 4.66 ± 0.15^c^ | 1.50 ± 0.09^c^ |  | \ | \ |
| 0.8 | −8.36 ± 0.15^d^ | −11.53 ± 0.15^e^ |  | 19.03 ± 0.11^b^ | 14.20 ± 0.09^b^ |  | 5.90 ± 0.20^b^ | 2.60 ± 0.26^b^ |  | \ | \ |
| 1.0 | −11.70 ± 0.20^e^ | −14.20 ± 0.09^f^ |  | 19.93 ± 0.15^a^ | 14.93 ± 0.35^a^ |  | 6.93 ± 0.15^a^ | 3.96 ± 0.20^a^ |  | \ | \ |

Different letters mean the significant difference (*P* < 0.05).

Table S7. *T_PAR_* of 3-mm-thickness organo-hydrocolloids with adding four sodium salts.

| Concentration (mol/L) | NaCl | | |  | NaI | | |  | NaBr | | |  | Na_2_SO_4_ | | |
| --- | --- | --- | --- | --- | --- | --- | --- | --- | --- | --- | --- | --- | --- | --- | --- |
|  | Transparent | −15°C (%) | 80°C (%) |  | Transparent | −15°C (%) | 80°C (%) |  | Transparent | −15°C (%) | 80°C (%) |  | Transparent | −15°C (%) | 80°C (%) |
| 0 | 90.90 ± 1.31^a^ | 3.38 ± 0.32^a^ | 9.19 ± 0.65^ab^ |  | 90.90 ± 1.31^a^ | 3.38 ± 0.32^a^ | 9.19 ± 0.65^a^ |  | 90.90 ± 1.31^a^ | 3.38 ± 0.32^a^ | 9.19 ± 0.65^a^ |  | 90.90 ± 1.31^a^ | 3.38 ± 0.32^a^ | 9.19 ± 0.65^a^ |
| 0.2 | 90.70 ± 1.01^a^ | 3.32 ± 0.13^a^ | 9.37 ± 0.18^a^ |  | 91.15 ± 1.61^a^ | 3.29 ± 0.15^a^ | 9.41 ± 0.60^a^ |  | 91.19 ± 1.18^a^ | 3.29 ± 0.16^a^ | 9.12 ± 0.58^a^ |  | 86.74 ± 1.01^b^ | 3.06 ± 0.16^a^ | 8.71 ± 0.51^a^ |
| 0.4 | 90.79 ± 1.21^a^ | 2.90 ± 0.15^b^ | 8.39 ± 0.56^b^ |  | 90.75 ± 1.31^a^ | 3.07 ± 0.32^ab^ | 9.57 ± 0.58^a^ |  | 89.79 ± 1.02^a^ | 2.74 ± 0.21^b^ | 8.86 ± 0.54^ab^ |  | \^c^ | \ | \ |
| 0.6 | 89.55 ± 0.98^a^ | 2.86 ± 0.21^b^ | 8.38 ± 0.72^b^ |  | 89.29 ± 0.98^a^ | 2.61 ± 0.21^b^ | 8.72 ± 0.51^a^ |  | 90.01 ± 1.16^a^ | 2.78 ± 0.22^b^ | 9.02 ± 0.36^a^ |  | \ | \ | \ |
| 0.8 | 58.09 ± 1.12^b^ | 2.84 ± 0.22^b^ | 8.36 ± 0.82b^b^ |  | 90.51 ± 0.98^a^ | 2.68 ± 0.16^b^ | 9.23 ± 0.56^a^ |  | 90.78 ± 1.09^a^ | 2.69 ± 0.18^b^ | 7.94 ± 0.58^b^ |  | \ | \ | \ |
| 1.0 | 52.28 ± 1.01^c^ | 2.85 ± 0.32^b^ | 7.01 ± 0.39^c^ |  | 83.74 ± 1.12^b^ | 2.59 ± 0.26^b^ | 8.44 ± 0.31^b^ |  | 75.30 ± 1.32^b^ | 2.64 ± 0.19^b^ | 7.45 ± 0.71^b^ |  | \ | \ | \ |

Different letters mean the significant difference (*P* < 0.05).

Table S8. *T_PAR_* of 3-mm-thickness organo-hydrocolloids with adding four potassium salts.

| Concentration (mol/L) | KCl | | |  | KI | | |  | KBr | | |  | K_2_SO_4_ | | |
| --- | --- | --- | --- | --- | --- | --- | --- | --- | --- | --- | --- | --- | --- | --- | --- |
|  | Transparent | −15°C (%) | 80°C (%) |  | Transparent | −15°C (%) | 80°C (%) |  | Transparent | −15°C (%) | 80°C (%) |  | Transparent | −15°C (%) | 80°C (%) |
| 0 | 90.90 ± 1.31^a^ | 3.38 ± 0.32^a^ | 9.19 ± 0.65^a^ |  | 90.90 ± 1.31^a^ | 3.38 ± 0.32^a^ | 9.19 ± 0.65^a^ |  | 90.90 ± 1.31^a^ | 3.38 ± 0.32^a^ | 9.19 ± 0.65^a^ |  | 90.90 ± 1.31^a^ | 3.38 ± 0.32^a^ | 9.19 ± 0.65^a^ |
| 0.2 | 90.99 ± 1.02^a^ | 3.36 ± 0.31^a^ | 9.01 ± 0.42^a^ |  | 90.76 ± 1.21^a^ | 3.77 ± 0.58^a^ | 9.01 ± 0.60^a^ |  | 90.95 ± 1.03^a^ | 2.81 ± 0.53^b^ | 9.12 ± 0.60^a^ |  | 86.50 ± 1.23^b^ | 3.15 ± 0.32^a^ | 8.15 ± 0.56^a^ |
| 0.4 | 91.12 ± 1.42^a^ | 3.07 ± 0.27^ab^ | 9.03 ± 0.41^a^ |  | 91.19 ± 1.02^a^ | 3.45 ± 0.23^a^ | 9.05 ± 0.54^a^ |  | 91.01 ± 1.09^a^ | 2.67 ± 0.16^b^ | 9.11 ± 0.59^a^ |  | \ | \ | \ |
| 0.6 | 90.84 ± 1.03^a^ | 2.90 ± 0.23^b^ | 8.01 ± 0.68^b^ |  | 91.22 ± 1.08^a^ | 3.27 ± 0.15^a^ | 9.02 ± 0.51^a^ |  | 90.99 ± 1.21^a^ | 2.56 ± 0.24^b^ | 9.03 ± 0.54^a^ |  | \ | \ | \ |
| 0.8 | 70.52 ± 1.06^b^ | 2.89 ± 0.21^b^ | 7.91 ± 0.51^b^ |  | 90.86 ± 1.29^a^ | 2.85 ± 0.11^b^ | 9.01 ± 0.50^a^ |  | 90.77 ± 1.11^a^ | 2.46 ± 0.25^b^ | 9.01 ± 0.94^a^ |  | \ | \ | \ |
| 1.0 | 61.97 ± 0.97^c^ | 3.10 ± 0.34^ab^ | 7.44 ± 0.53^b^ |  | 84.19 ± 1.21^b^ | 2.64 ± 0.35^b^ | 9.11 ± 0.52^a^ |  | 78.13 ± 1.18^b^ | 2.51 ± 0.24^b^ | 7.17 ± 0.88^b^ |  | \ | \ | \ |

Different letters mean the significant difference (*P* < 0.05).


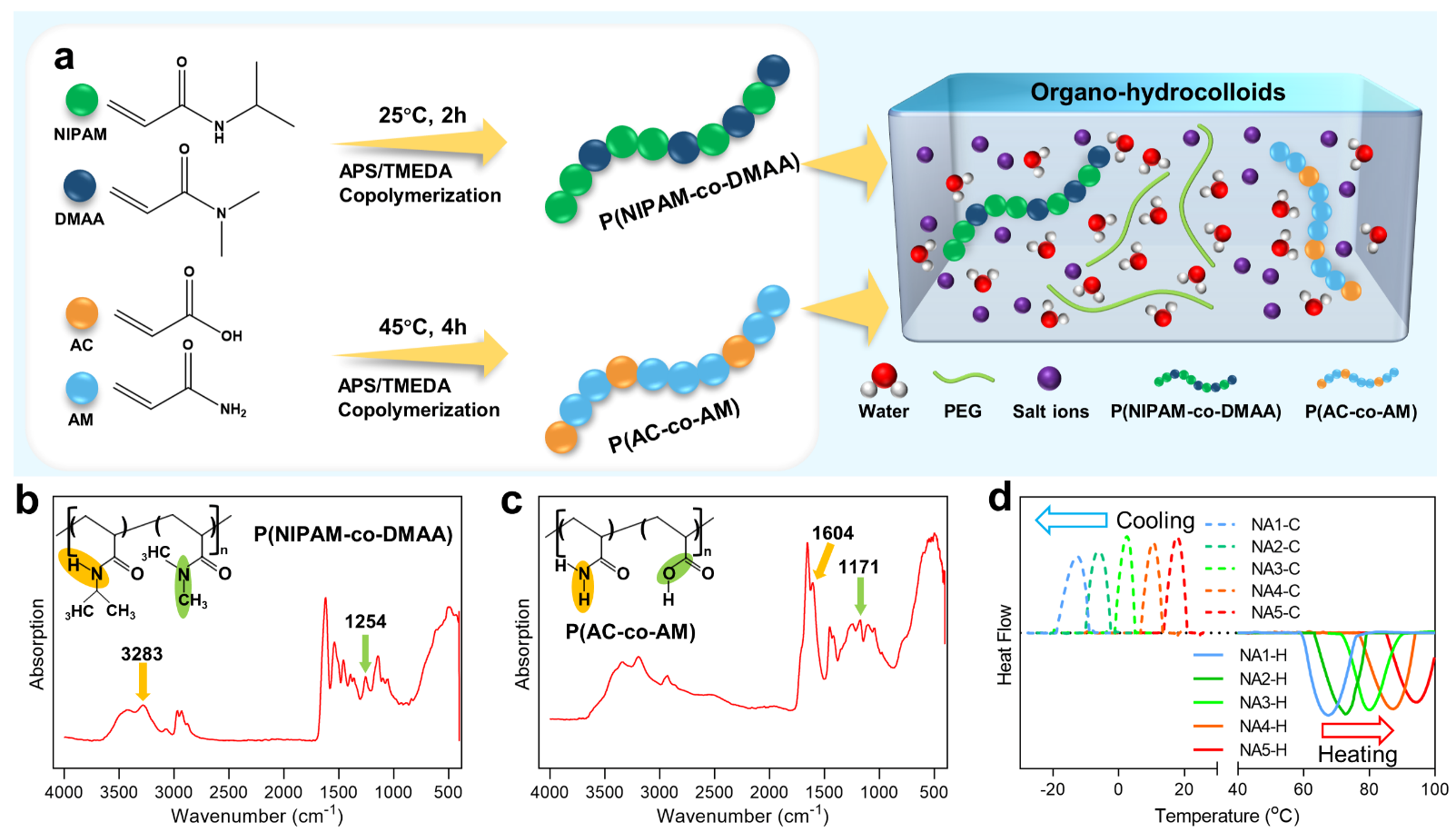


Figure S1 Synthesis and FTIR spectra of copolymers. (a) Chemical synthesis pathway and colloidal schematic diagram of p(NIPAM-co-DMAA) and p(AC-co-AM), respectively. (b) N–H of NIPAM and N–C of DMAA in p(NIPAM-co-DMAA) appear at 3283 and 1254 cm^−1^, respectively. (c) C–O of AC and N–H of AM in p(AC-co-AM) appear at 1171 and 1604 cm^−1^, respectively. (d) The DSC curves of copolymers after mixing. NA1-C and NA1-H represent the copolymers with UCST and LCST in NA1 organo-hydrocolloids.


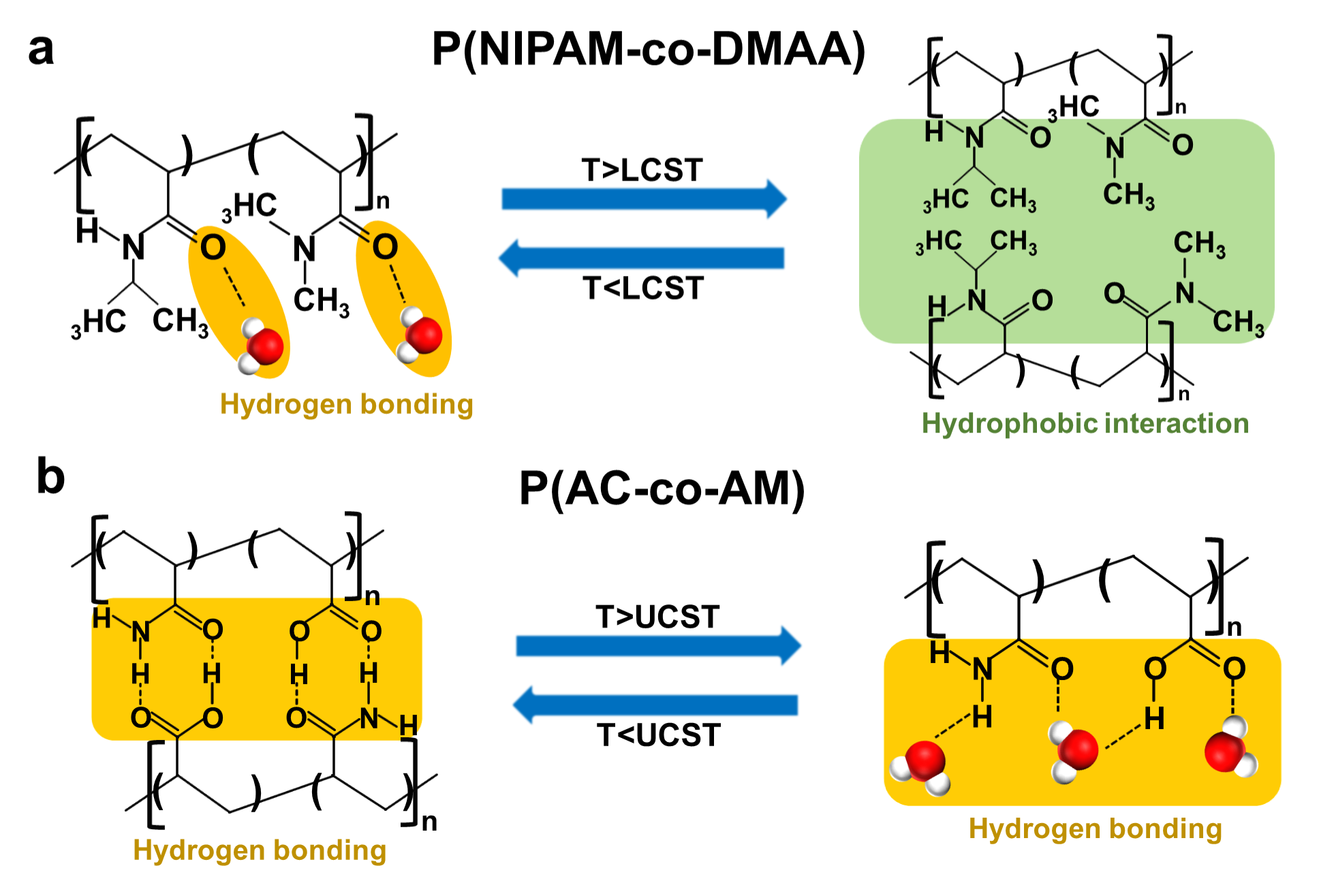


Figure S2 Molecular mechanisms of thermochromism. (a) P(NIPAM-co-DMAA) can hydrogen bond with water when T<LCST, while the hydrophobic interaction will interrupt the hydrogen bonding when T>LCST. (b) P(AC-co-AM) can hydrogen bond with water when T>UCST and hydrogen bond within the copolymer chain when T<UCST.


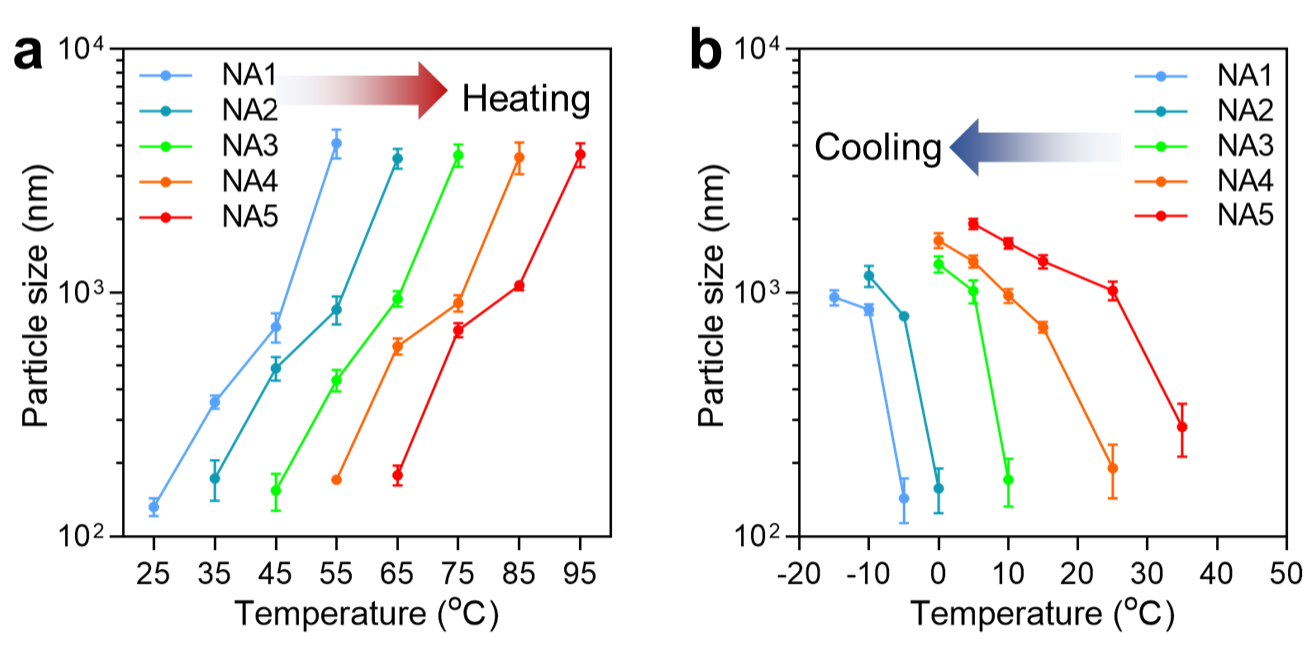


Figure S3 Particle sizes of aggregates generated in nanocolloid systems. (a) Heat-induced thermochromism makes p(NIPAM-co-DMAA) aggregate by hydrophobic interaction, generating particles as temperature rises. (b) Cold-induced thermochromism makes p(AC-co-AM) aggregate by intramolecular hydrogen bonding, generating particles as temperature reduces.


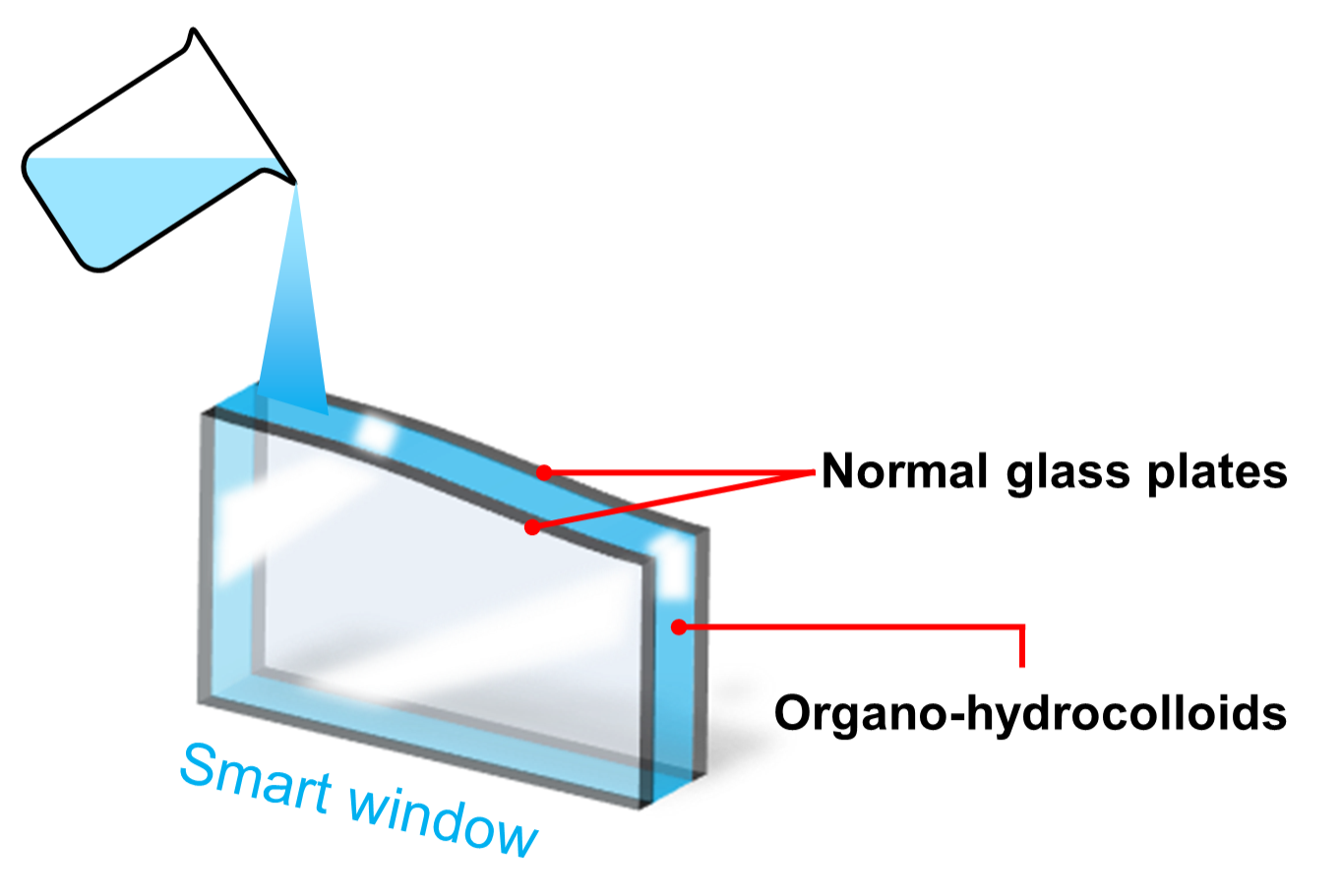


Figure S4 Fabrication of the smart window. Schematic diagram of making smart windows using liquid organo-hydrocolloids.


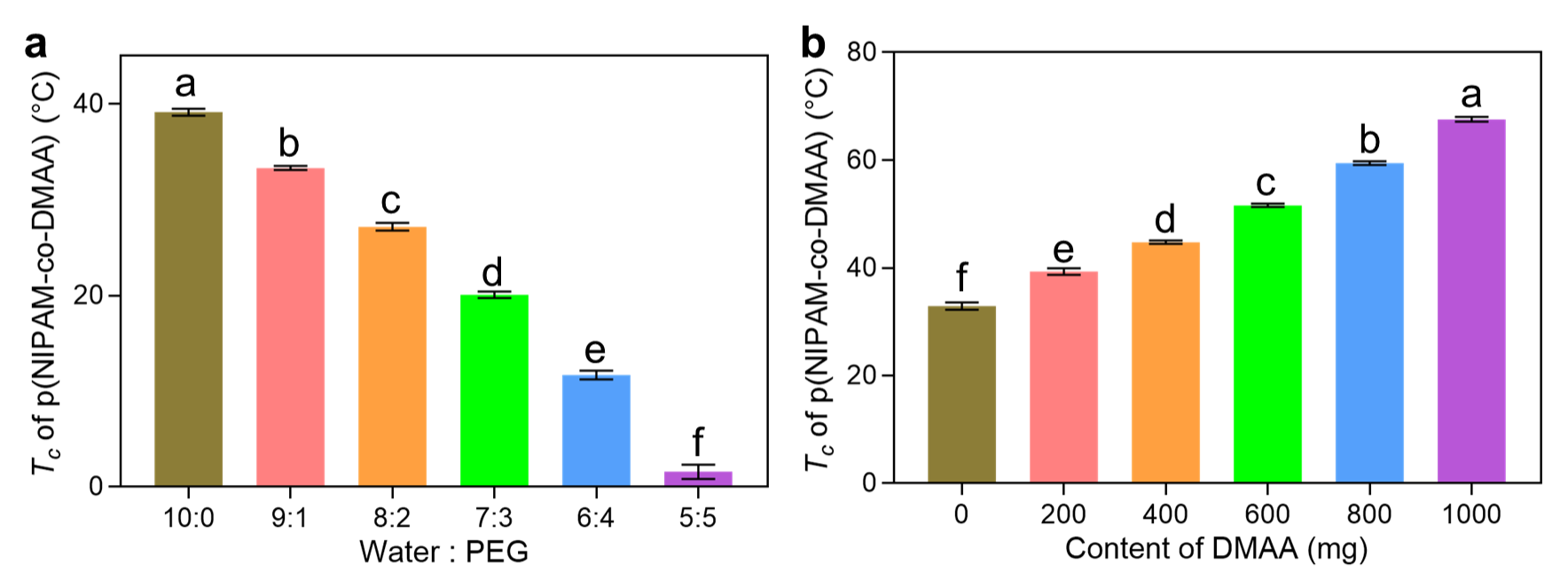


Figure S5 Effects of the PEG and the DMAA on the *T_c_* of p(NIPAM-co-DMAA). (a) By increasing PEG from 0 to 50% (vol.), the *T_c_* of p(NIPAM-co-DMAA) (synthesized by 800 mg of NIPAM and 200 mg of DMAA) significantly decreases from 39.1 to 1.6 °C. (b) By increasing DMAA from 0 to 1000 mg for synthesizing p(NIPAM-co-DMAA) with 800 mg of NIPAM, the *T_c_* of p(NIPAM-co-DMAA) increases from 33 to 67 °C.


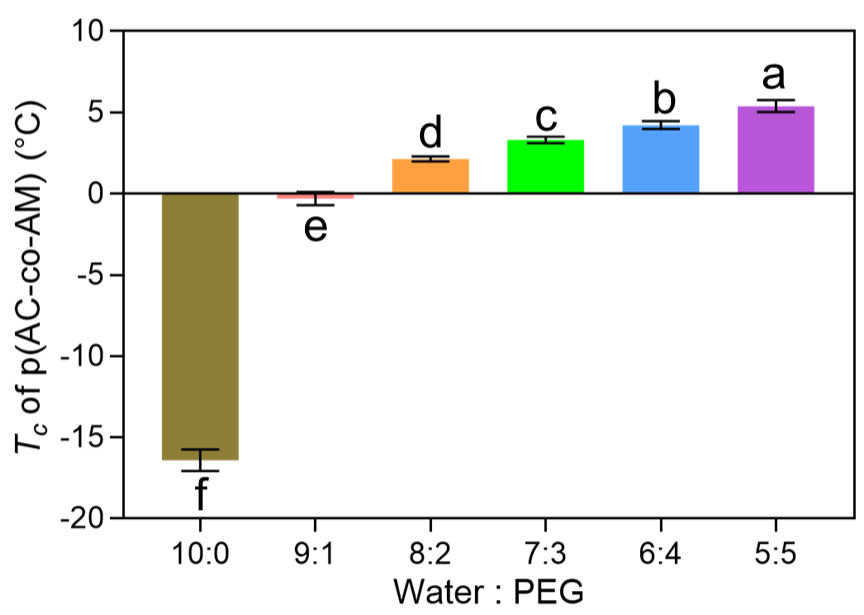


Figure S6 The effect of the PEG on the *T_c_* of p(AC-co-AM). By increasing the PEG from 0 to 50% (vol.), the *T_c_* of p(AC-co-AM) (synthesized by 300 mg of AC and 1000 mg of AM) increases from −16 to 5.4 °C.


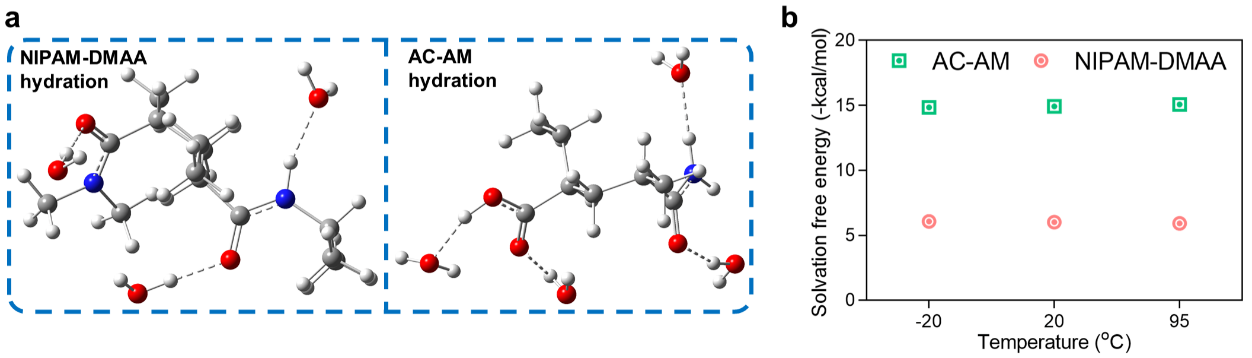


Figure S7 DFT calculation of solvation free energy. (a) Optimized molecular models of hydrated structures between sequences (NIPAM-DMAA and AC-AM) and water molecules. (b) At −20, 20, and 95 °C, the solvation free energy of AC-AM is higher than that of NIPAM-DMAA.


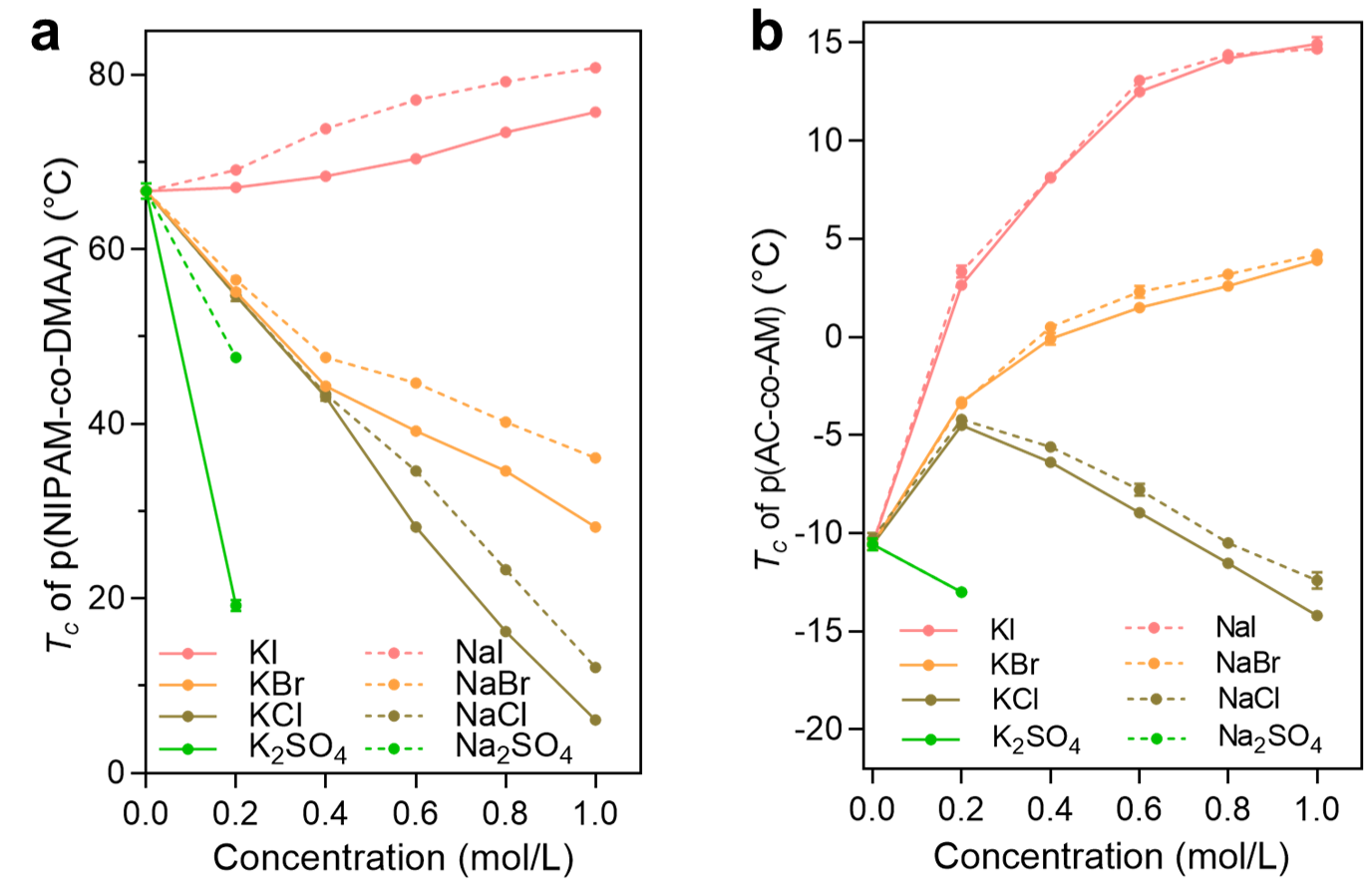


Figure S8 Effects of salts on the *T_c_* of organo-hydrocolloids. Impacts of eight salts with different concentrations on the *T_c_* of (a) heat-thermochromic p(NIPAM-co-DMAA) and (b) cold-thermochromic p(AC-co-AM). Detailed data are listed in Tables S3–S6.


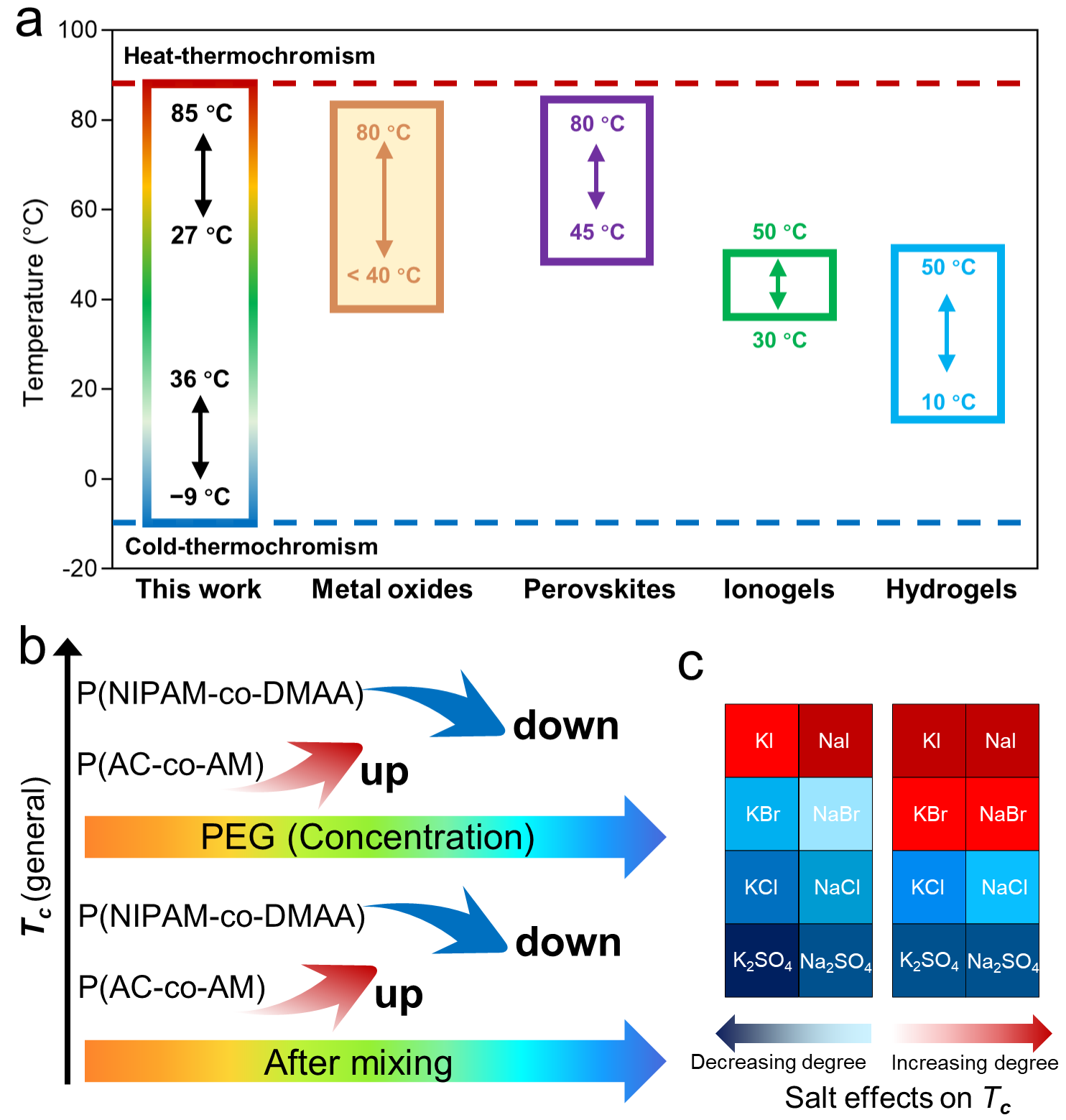


Figure S9 A summary of thermochromic materials for smart windows (a). Our organo-hydrocolloids have bidirectional thermochromism and a wide range of temperature adjustments. The corresponding comparative materials are from the main text references [9b], [30], [10], [12b], [31], [32], and [33]. A schematic diagram showing the overall impact of PEG concentration, the mixing effect (b), and salt effects (c) on the phase transition temperature.


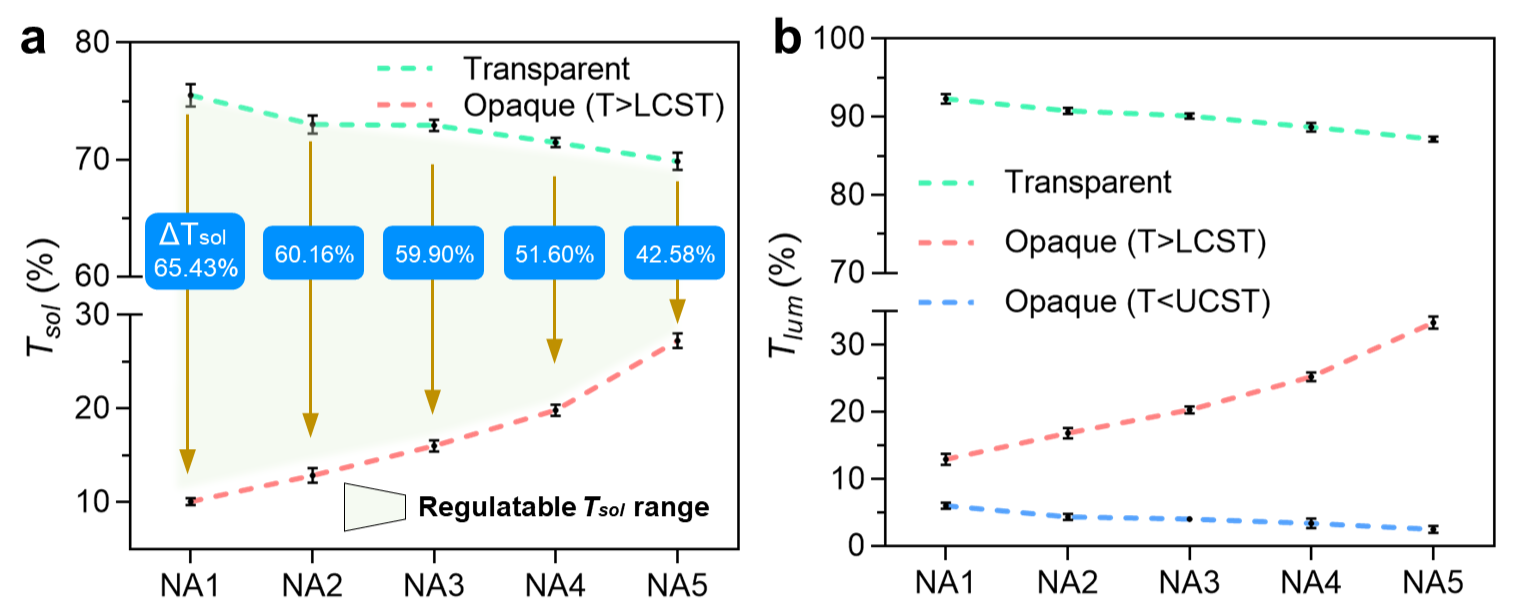


Figure S10 Calculated *T_sol_* and *T_lum_* of smart windows. (a) From NA1 to NA5, Smart windows show a decrease of *T_sol_* at the transparent state, an increase of *T_sol_* at the opaque state, and an attenuated *ΔT_sol_*. (b) The regulation of *T_lum_* in cold-induced thermochromism is enhanced from NA1 to NA5, which is opposite to that of heat-induced thermochromism.


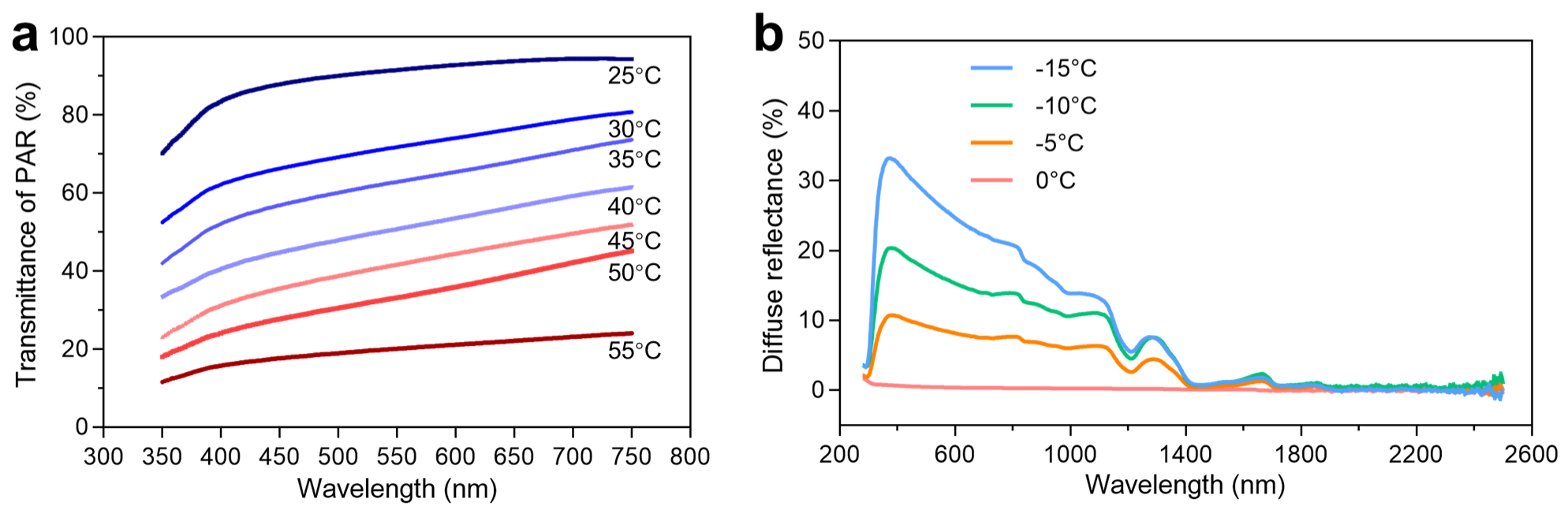


Figure S11 Transmittance and diffuse reflectance at different temperatures. (a) As the temperature increases from 25 to 55 °C, the transmittance of the 3-mm-thickness NA1 smart window continuously decreases between 350 to 750 nm. (b) As the temperature decreases from 0 to −15 °C, the diffuse reflectance of the 3-mm-thickness NA1 smart window continuously increases between visible light and NIR.


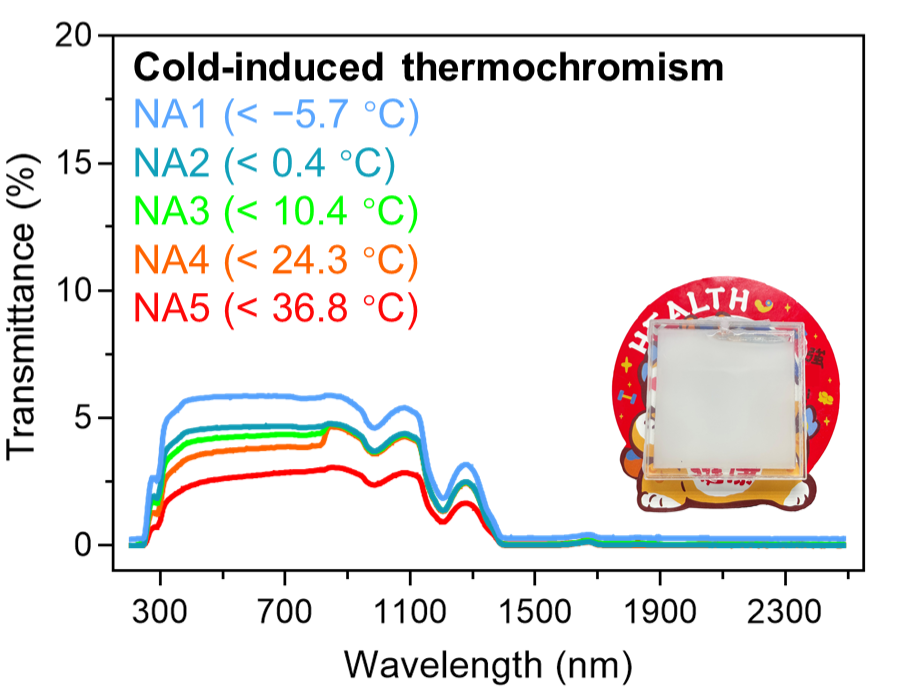


Figure S12 Transmittance of cold-thermochromic smart windows. After complete cold-induced thermochromism, the transmittance of 3-mm-thickness smart windows based on different organo-hydrocolloids gradually decreases from NA1 to NA5 (visible light and NIR).


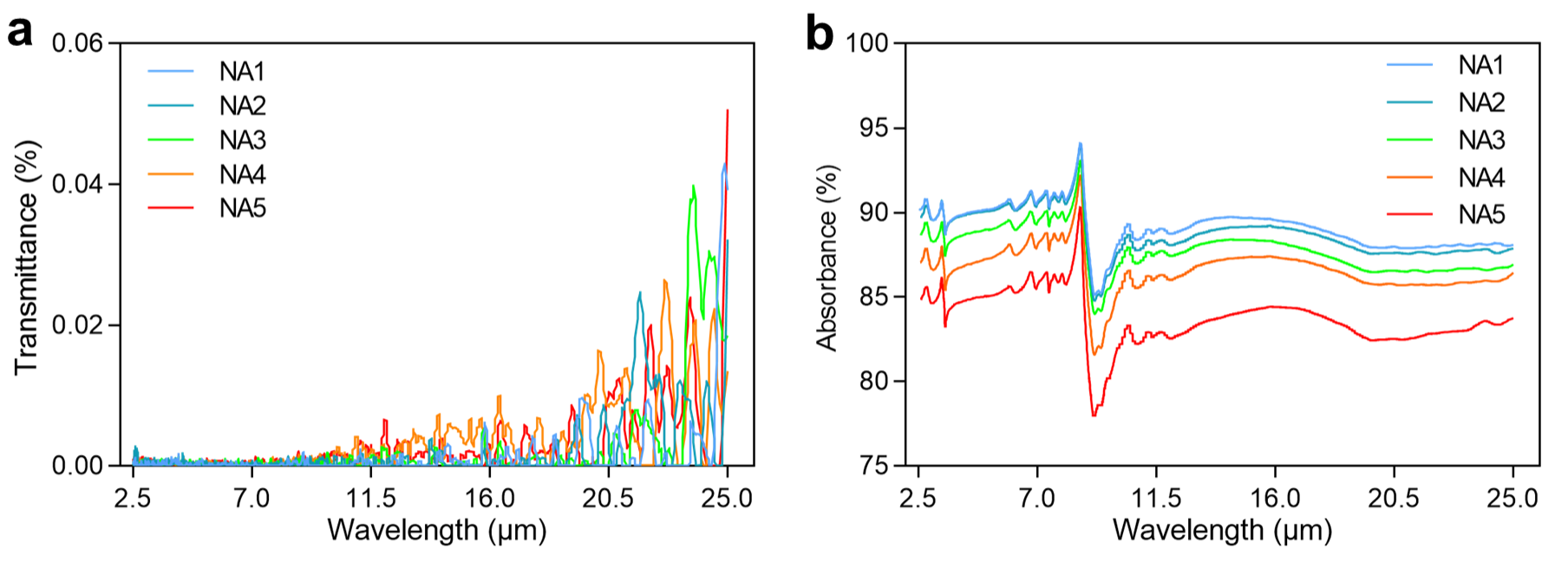


Figure S13 Transmittance and absorbance of smart windows in mid-infrared. After cold-induced thermochromism, (a) the mid-infrared transmittance of smart windows is very low, (b) which is related to the high absorbance and diffuse reflection of the mid-infrared by smart windows.


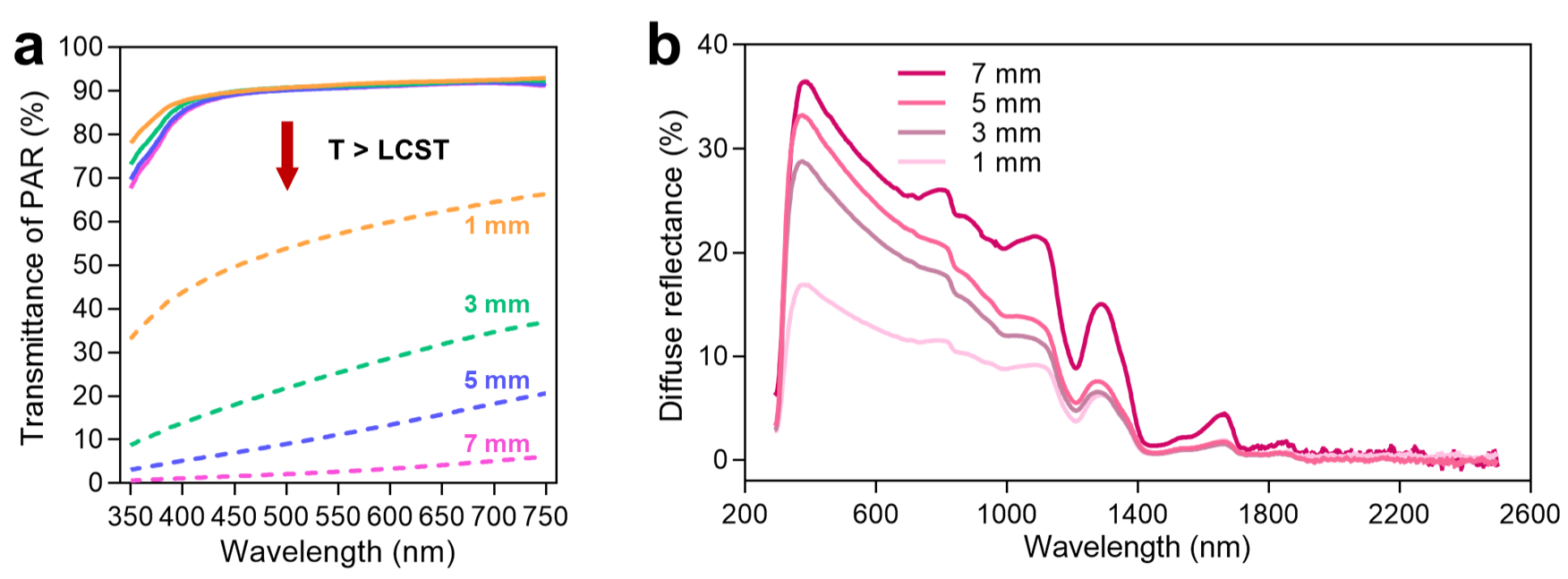


Figure S14 Transmittance and diffuse reflectance at different thicknesses. (a) Thickness slightly affects the transparent state of smart windows but significantly decreases the transmittance of PAR as the thickness increases. (b) As the thickness increases, the diffuse reflectance of smart windows is gradually enhanced.


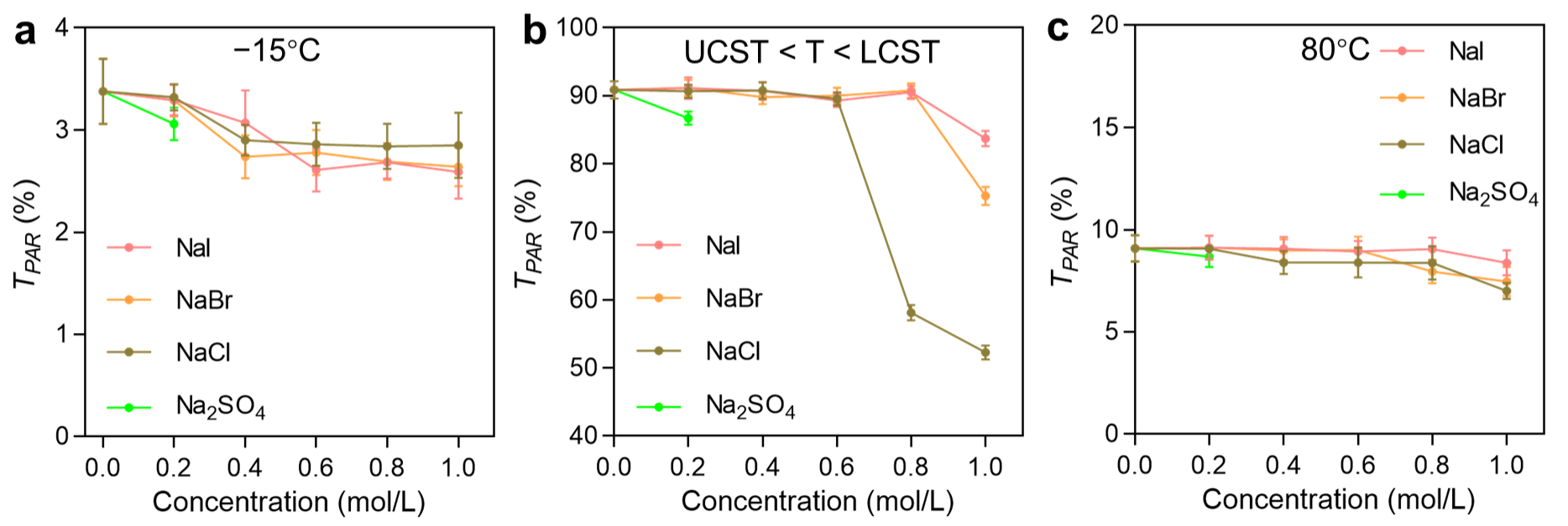


Figure S15 *T_PAR_* of organo-hydrocolloids with four sodium salts. (a) By cold-induced thermochromism, an increase in salt concentrations slightly reduces *T_PAR_*, which is not affected by the type of salt. (b) At the transparent state, salt concentrations close to saturation enhance the interaction among copolymers, leading to a decrease in *T_PAR_*. (c) By heat-induced thermochromism, high salt concentrations slightly reduce *T_PAR_*. However, the type of salt is not important in regulating *T_PAR_*.


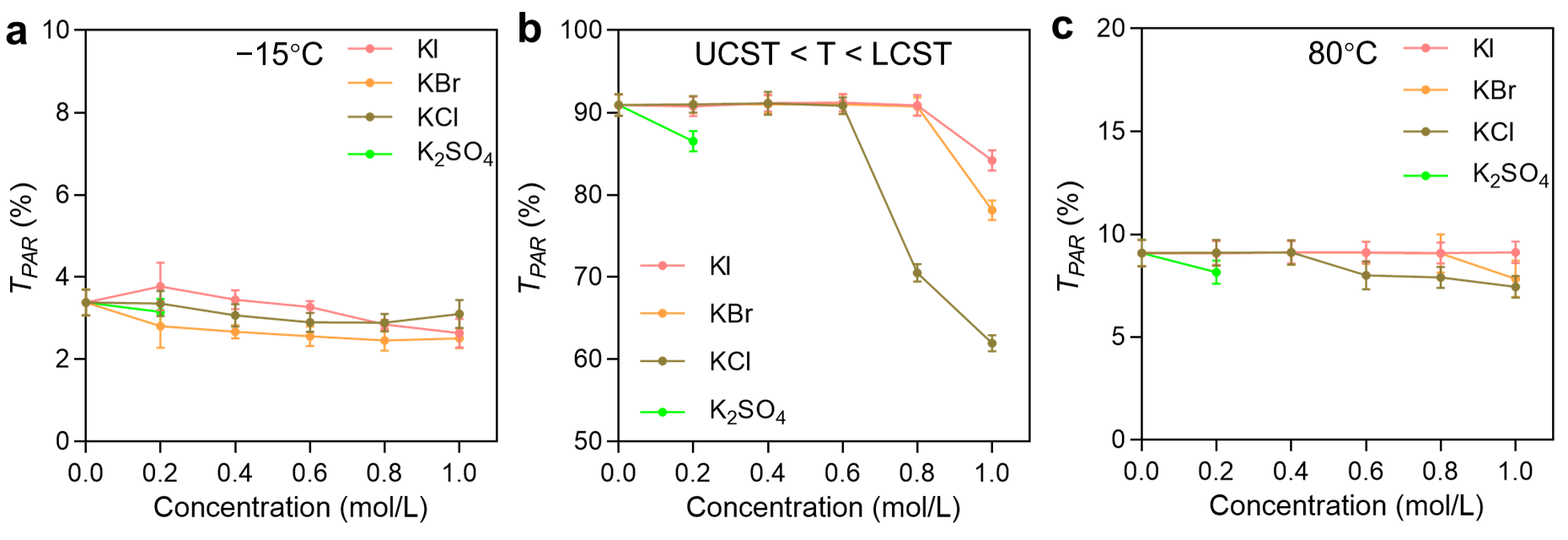


Figure S16 *T_PAR_* of organo-hydrocolloids with four potassium salts. The impact of potassium salts on *T_PAR_* is similar to that of sodium salts, illustrating that high salt concentrations should be avoided to cause undesirable loss of optical performances of organo-hydrocolloids.


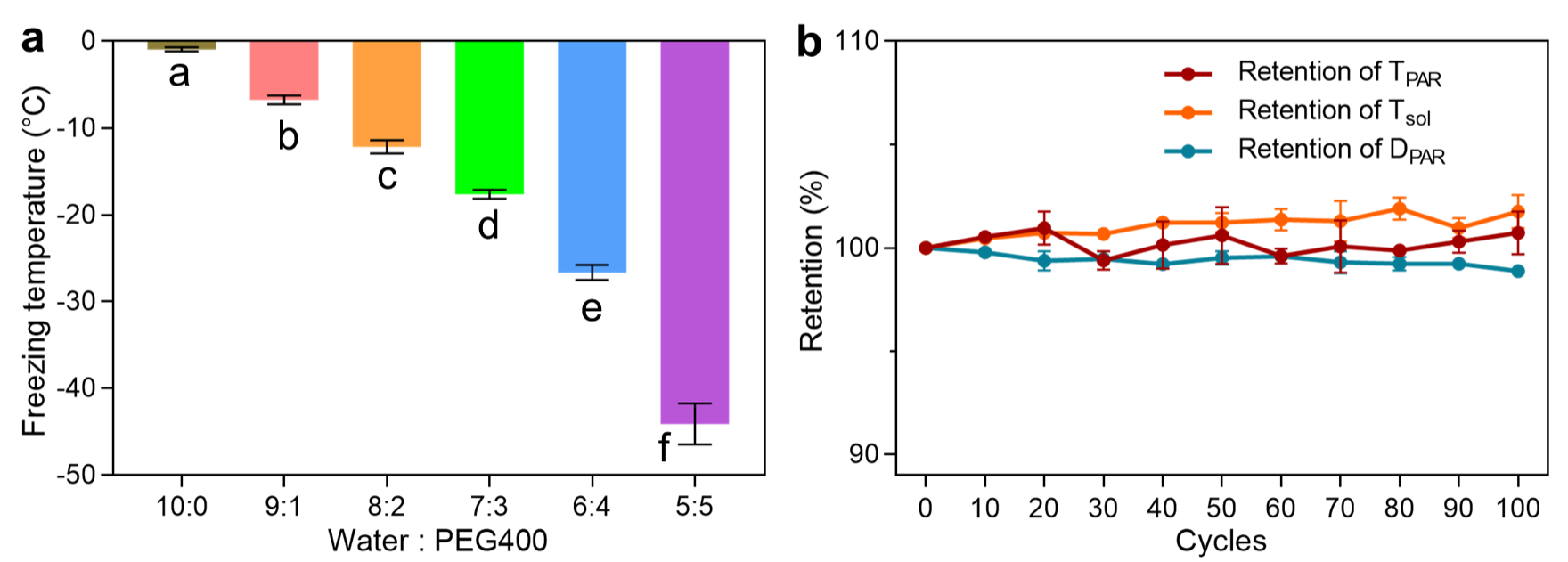


Figure S17 Environmental stability and reusability of organo-hydrocolloid-based smart windows. (a) The freezing point of NA1 colloids in mediums with the PEG from 0 to 50% (vol.) significantly decreases from −0.93 to −44.1 °C. (b) The retention of *T_PAR_*, *T_sol_*, and *D_PAR_* of the NA1-based smart window during the temperature cycles displays a fluctuation amplitude of less than 3%. Different lowercase letters below the bars in (a) indicate significant differences (*P* < 0.05).


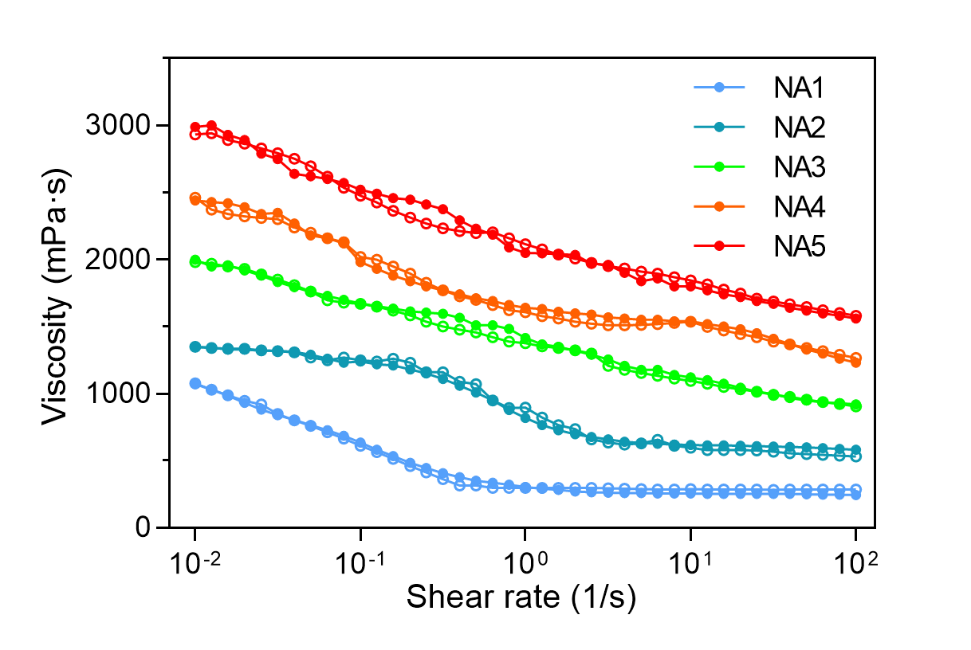


Figure S18 Viscosity of organo-hydrocolloids tested by a rheometer. All organo-hydrocolloids exhibit the characteristic of shear-thinning; as the monomer concentration increases, the viscosity of organo-hydrocolloids continues to increase. The high viscosity is important in preventing the settling of particles formed by the aggregation of copolymers. The curves connected be hollow circles represent the viscosity of the corresponding organo-hydrocolloids after 6 months of outdoor operation, showing no significant changes.


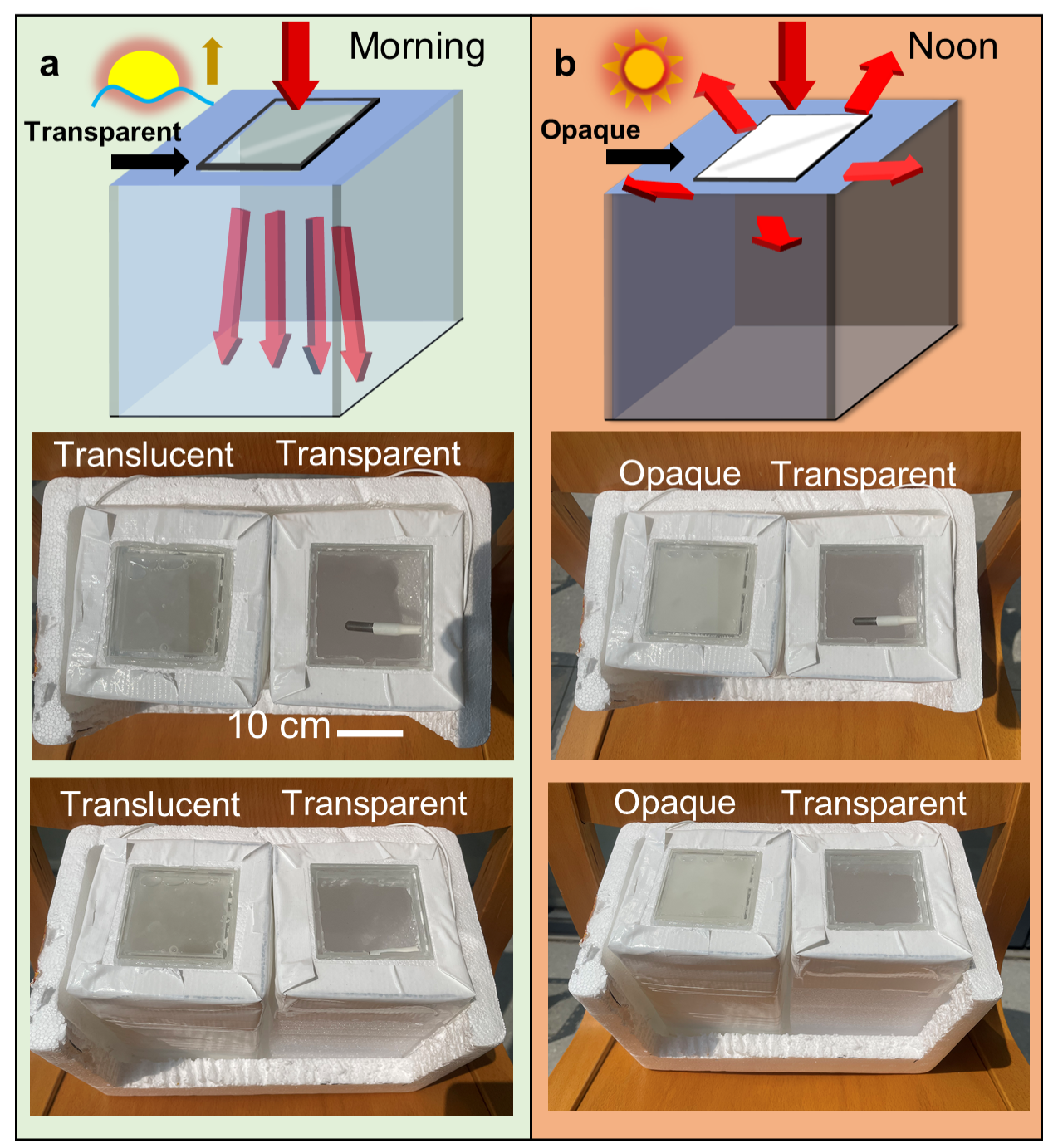


Figure S19 Energy saving simulation of smart windows in a hot summer. (a) In the morning, the NA1-based smart window is transparent or translucent, allowing partial solar radiation to pass through. (b) At noon, the smart window becomes opaque to prevent sunlight from passing, thus cooling the internal space.


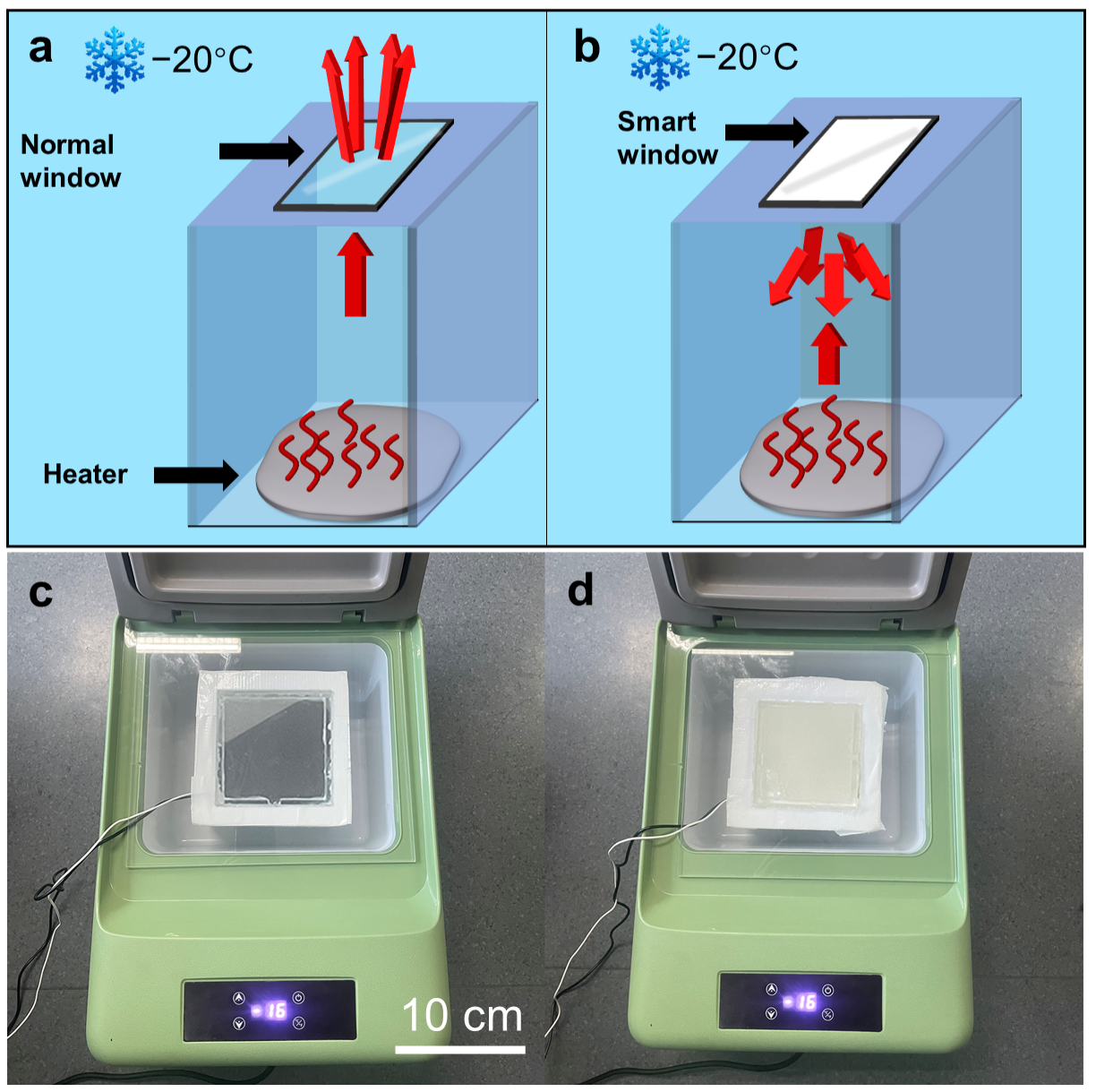


Figure S20 Energy saving simulation of smart windows in a cold environment. The thermal radiation from the heater (5 W) radiates outside the box covered with (a) the normal window but cycles inside the box covered with (b) the smart window. (c) and (d) Photos of boxes covered with the normal and smart windows in the cold environment at −20 °C.


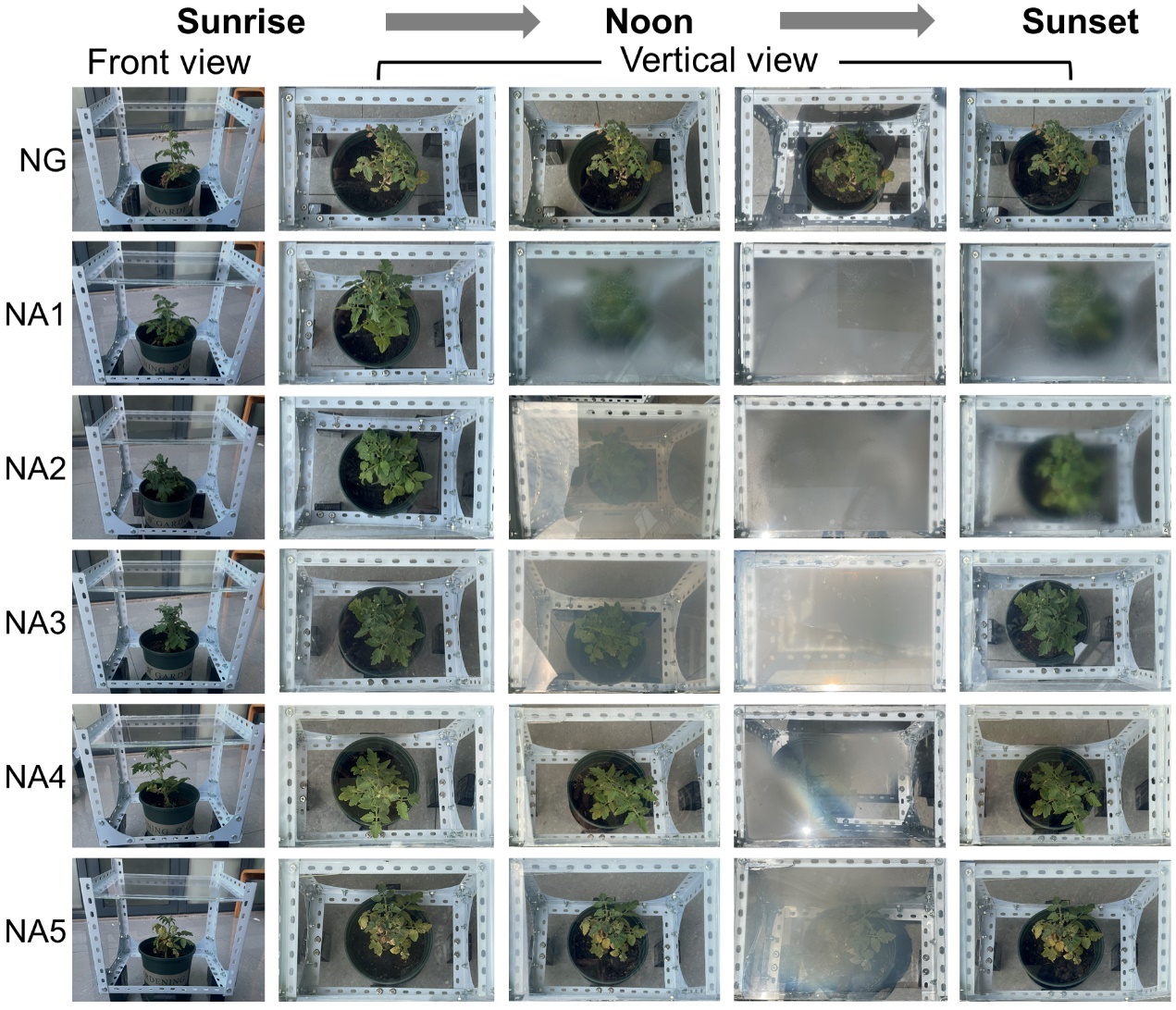


Figure S21 Photos of simulated indoor farming under heat stress. From sunrise to sunset, the opaque duration of smart windows is shortened from NA1 to NA5. At noon, NA1 has the lowest transmittance of solar radiation, and NA5 has the highest transmittance, meaning NA1 has a better cooling efficiency for tomato seedlings.


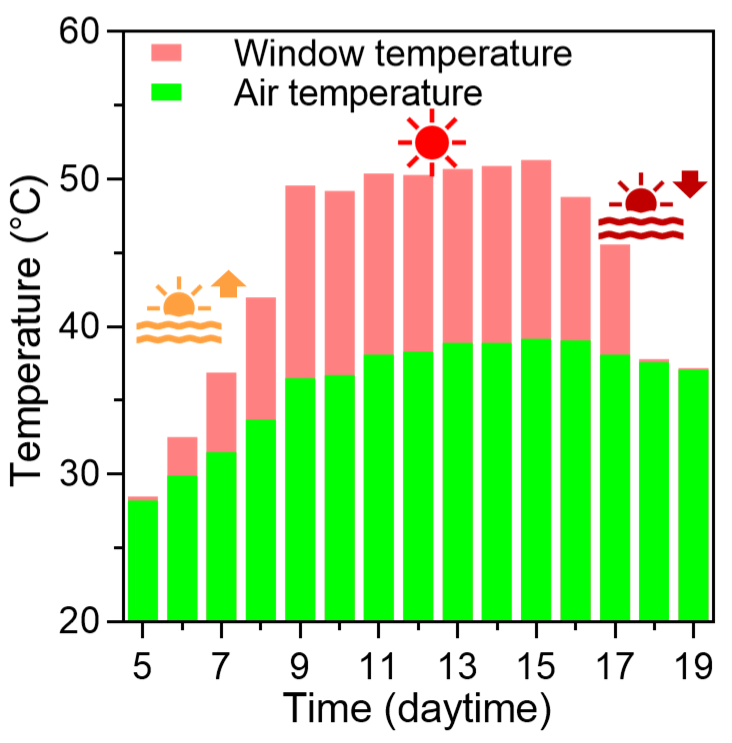


Figure S22 The temperature of simulated indoor farming under heat stress. Smart windows heated by sunlight show higher temperatures than air temperatures.


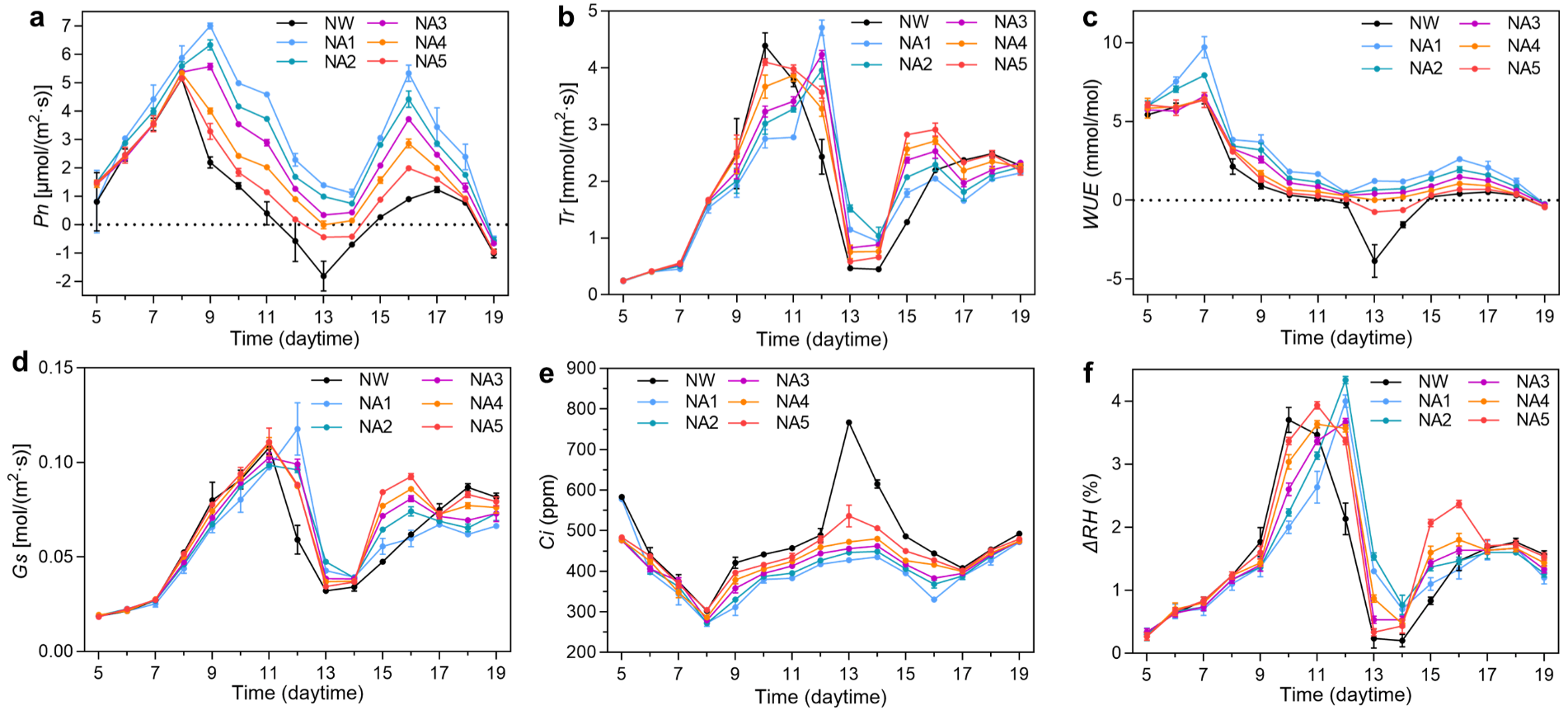


Figure S23 Complete physiological indicators of tomato seedlings covered by NA1 to NA5-based smart windows. The longer opaque time caused by smart windows makes the cooling effect of leaves more effective, suppressing stomata closure, increasing the activity of photosynthetic enzymes, improving water use efficiency, and thus increasing the photosynthetic rate of tomato seedlings.


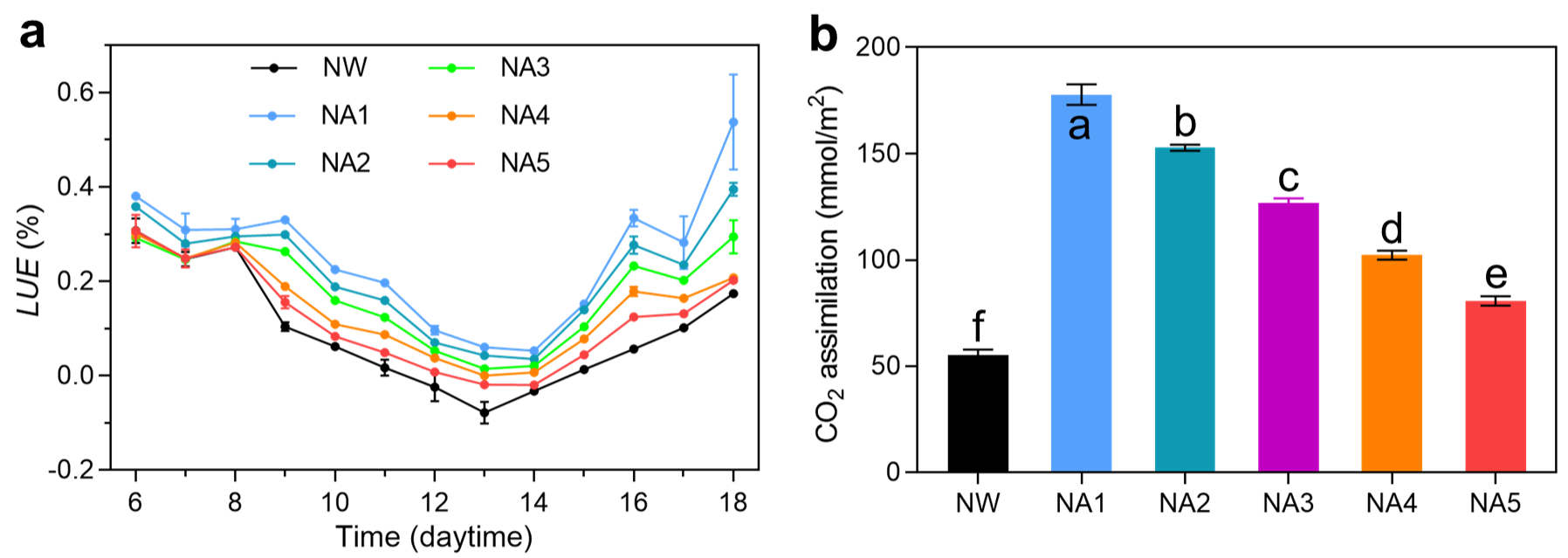


Figure S24 Complete *LUE* and CO_2_ assimilation of tomato seedlings. (a) In the hot summer, the stronger cooling effect of smart windows on tomato seedlings results in a higher *LUE*, (b) thereby increasing the CO_2_ assimilation of tomato seedlings to a greater extent. Different lowercase letters above the bars in (b) indicate significant differences (n = 10, *P* < 0.05).


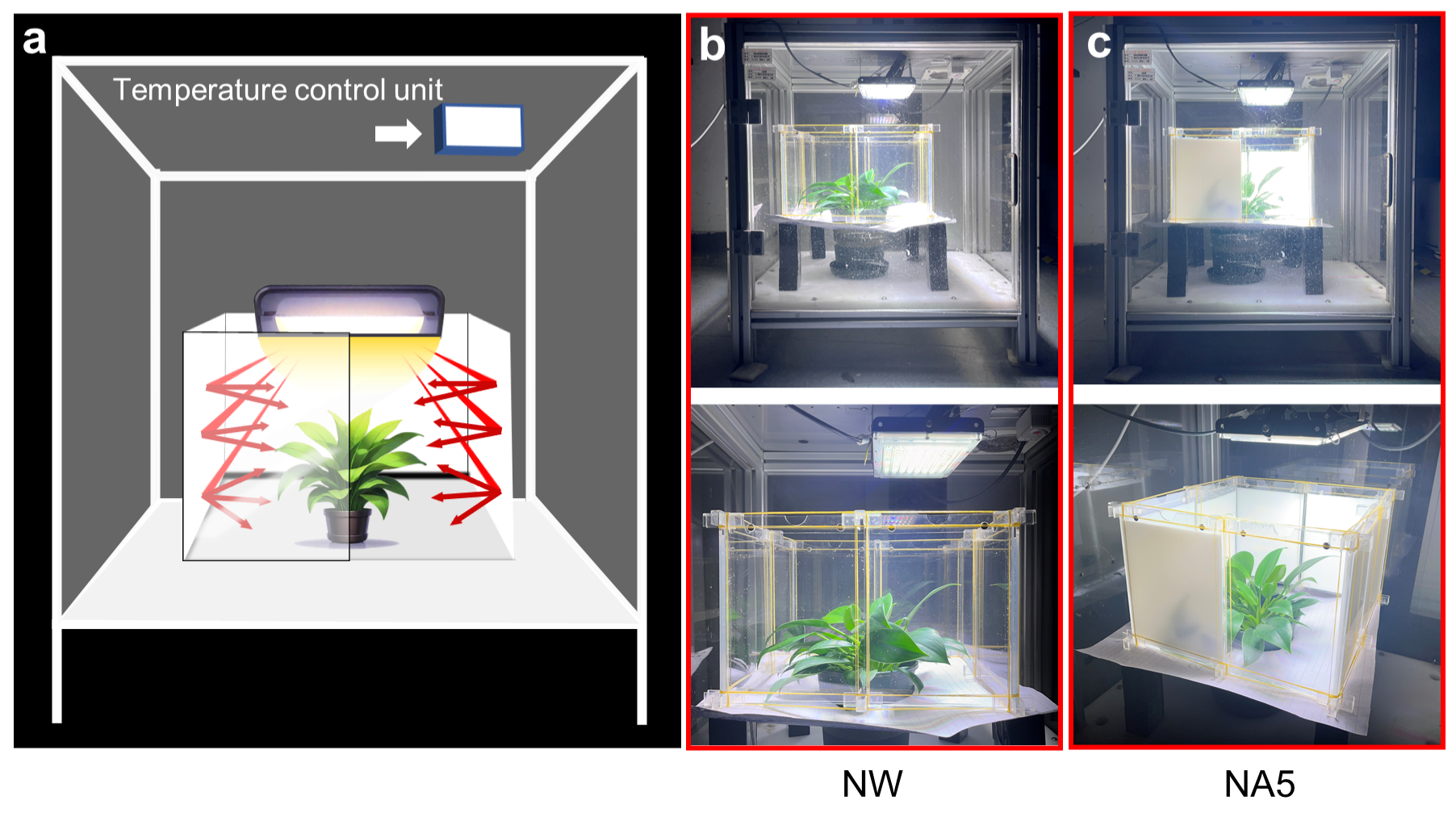


Figure S25 Photos of simulated indoor farming under cold stress. (a) In the environmental incubator with a ventilation device, the NA5-based smart window surrounds the *Epipremnum aureum* to form walls, and a supplementary lamp (100 W) with the full PAR range is placed above it. (b) When the ambient temperature is controlled at 35 °C, the smart window is transparent and does not reflect radiation. (c) When the temperature reaches 10 °C, the smart window becomes opaque and achieves diffuse reflection of PAR and more thermal radiation, increasing the leaf temperature and the PAR intensity that leaves can receive.


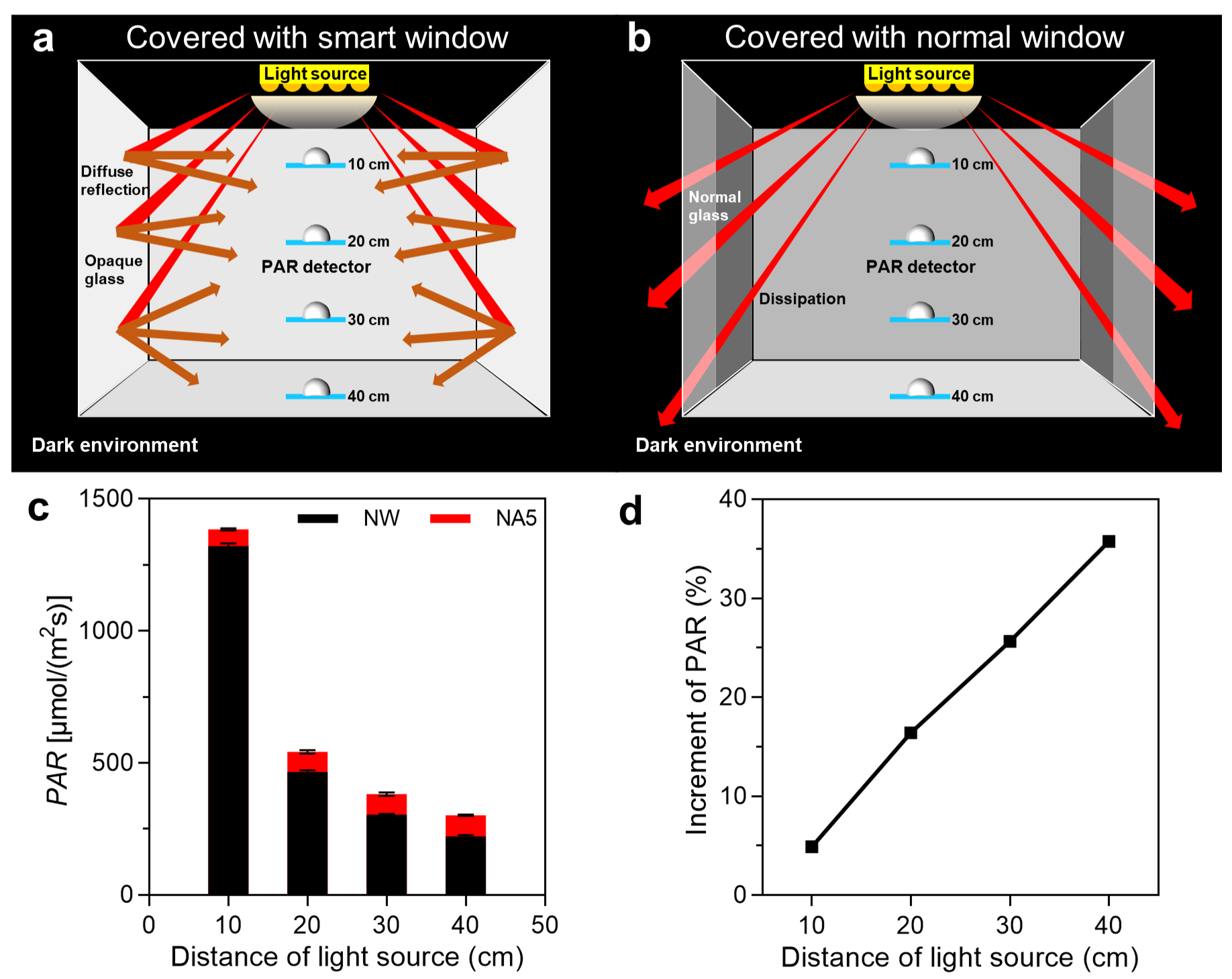


Figure S26 The relation between light source distance and PAR reflection. As the distance from the light source increases, the increase in the reflection area of the NA5-based smart window leads to a linear increment in the PAR intensity enhancement.


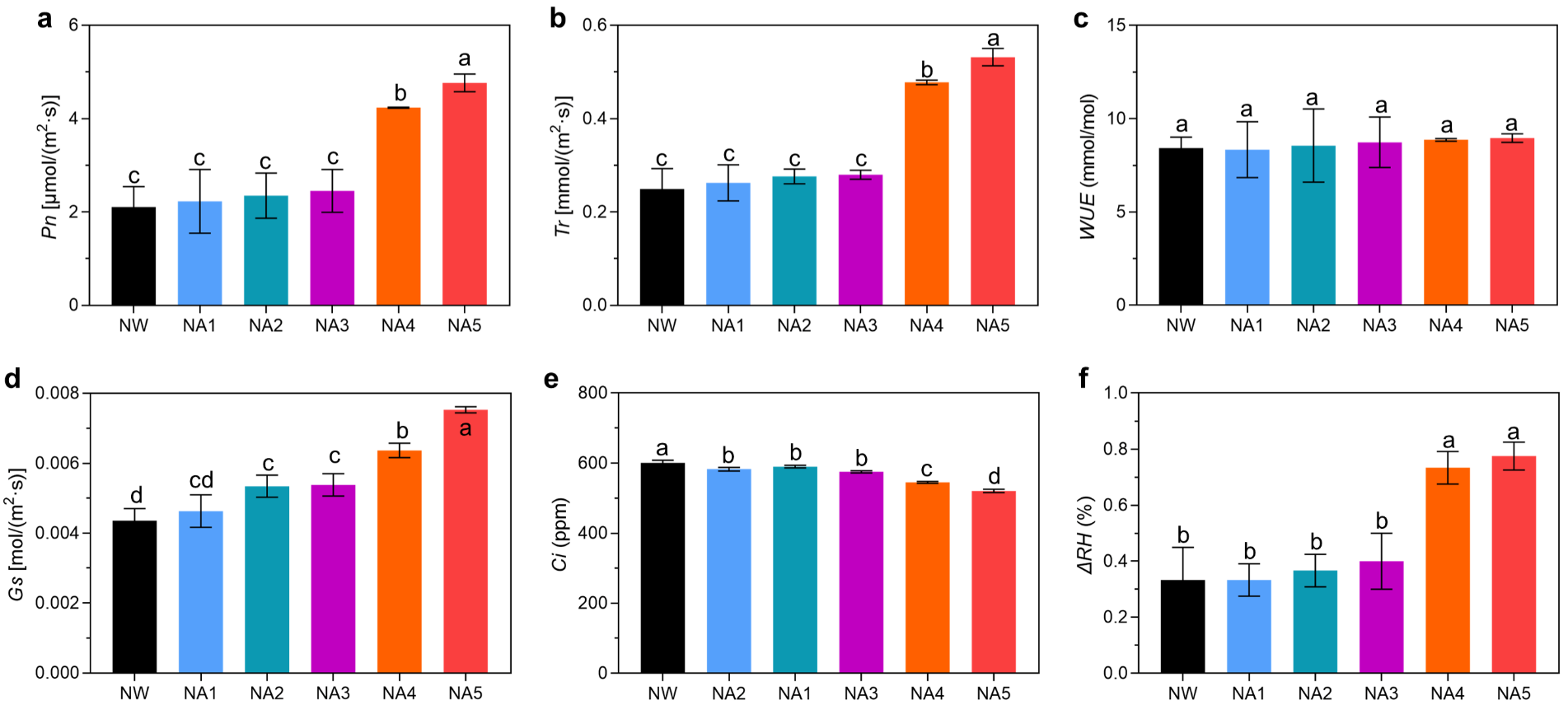


Figure S27 Complete physiological indicators of *Epipremnum aureum* covered by NA1 to NA5-based smart windows. The environmental temperature only allows for the cold-induced thermochromism of NA4 and NA5-based smart windows, resulting in only the NA4 and NA5 having an effect on increasing the leaf temperature and inhibiting stomata closure, thereby improving the photosynthetic rate of crops. Different lowercase letters above the bars in (a–f) indicate significant differences (n = 10, *P* < 0.05).


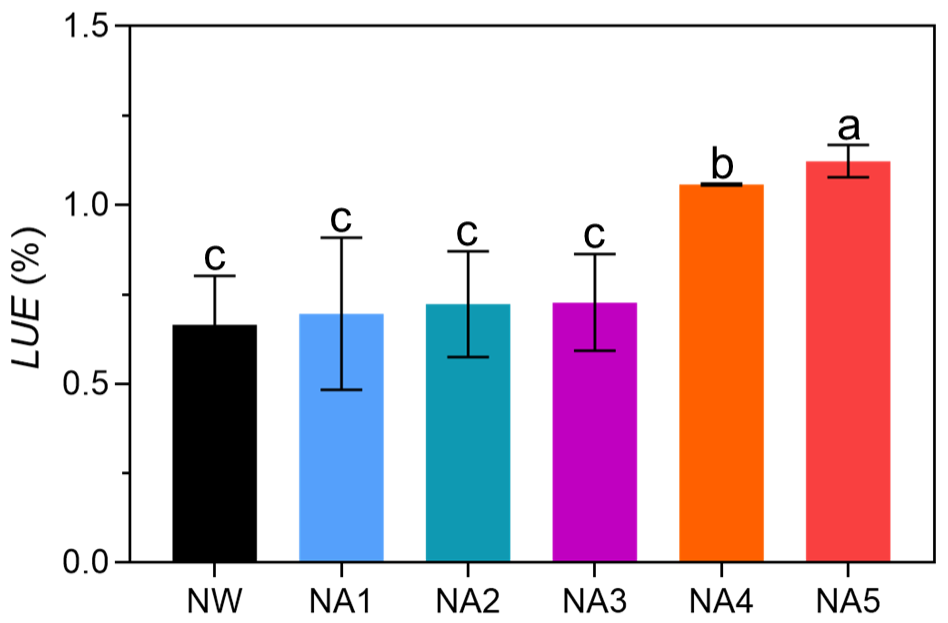


Figure S28 *LUE* of *Epipremnum aureum* surrounded by smart windows. Only the opaque NA4 and NA5 can improve the LUE of crops, which means that the diffuse reflection of smart windows has the potential to enhance crop overwintering cultivation and save electricity power for electro-agriculture. Different lowercase letters above the bars indicate significant differences (n = 10, *P* < 0.05).


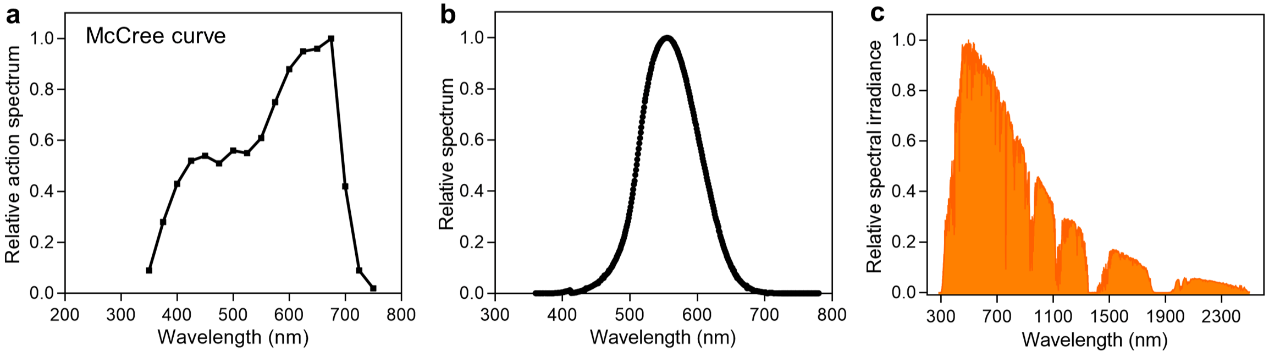


Figure S29 Standard curves for calculating *T_PAR_*, *D_PAR_*, *T_lum_*, and *T_sol_*. (a) The classic McCree absorption curve of crops in the PAR region (350–750 nm). (b) The spectral luminous efficiency function describes the visible perception of human eyes in the visible spectrum range (360–780 nm). (c) Spectral irradiance (250–2500 nm) of ASTM G173 measured at 37 ° tilted condition.
